# Accurate and interpretable prediction of chemical oxygen demand using explainable boosting algorithms with SHAP analysis

**DOI:** 10.1038/s41598-026-38757-4

**Published:** 2026-02-13

**Authors:** Khaled Merabet, Sungwon Kim, Salim Heddam, Fabio Di Nunno, Francesco Granata, Ozgur Kisi, Rana Muhammad Adnan, Mohammad Zounemat-Kermani, Christoph Külls

**Affiliations:** 1 Faculty of Science, Agronomy Department, Hydraulics Division, University 20 Août 1955 Skikda, Route El Hadaik, BP 26, Skikda, Algeria; 2 Department of Railroad Construction and Safety Engineering, Dongyang University, Yeongju, 36040 Republic of Korea; 3 Department of Civil and Mechanical Engineering (DICEM), University of Cassino and Southern Lazio, Via Di Biasio, 43, 03043 Cassino, Frosinone, Italy; 4 Department of Civil Engineering, Technische Hochschule Lübeck, 23562 Lübeck, Germany; 5 Department of Civil Engineering, School of Technology, IIia State University, 0162 Tbilisi, Georgia; 6 School of Civil, Environmental and Architectural Engineering, Korea University, Seoul, 02841 South Korea; 7 Department of Anatomy, Saveetha Medical College and Hospital, Saveetha Institute of Medical and Technical Sciences, Chennai, India; 8 College of Chemistry and Environmental Engineering, Water Science and Environmental Research Centre, Shenzhen University, Shenzhen, People’s Republic of China; 9 Department of Civil Engineering, Shahid Bahonar University of Kerman, Kerman, Iran

**Keywords:** COD prediction, Water quality, Boosting, Interpretability, SHAP, Chemistry, Environmental sciences

## Abstract

Accurate prediction of Chemical Oxygen Demand (COD) is vital for effective water quality management and pollution control. This study compares six ensemble boosting models, AdaBoost, CatBoost, XGBoost, LightGBM, HistGBRT, and NGBoost, for estimating COD from multiple water quality parameters, including pH, dissolved oxygen, suspended solids, and specific conductance. Data from two monitoring stations in South Korea (Toilchun and Hwangji) were used to train and validate the models. Model performance was evaluated using RMSE, MAE, R, NSE, and PBIAS, while interpretability was assessed through SHapley Additive exPlanations (SHAP). Results showed that NGBoost achieved the highest predictive accuracy at Toilchun (R = 0.979, NSE = 0.958, RMSE = 0.397 mg/L), while CatBoost performed best at Hwangji (R = 0.861, NSE = 0.733, RMSE = 0.477 mg/L). As NGBoost provides predictive probability distributions rather than single estimates, its results also reflect model uncertainty, supporting a more robust quantification of COD variability. SHAP analysis identified total organic carbon (TOC), biochemical oxygen demand (BOD₅), and suspended solids (SS) as the most influential variables controlling COD dynamics.

**Supplementary Information:**

The online version contains supplementary material available at 10.1038/s41598-026-38757-4.

## Introduction

The degradation of water quality is an increasingly urgent issue globally, affecting ecosystems, public health, and economic stability. COD, a key indicator of water pollution, measures the oxygen amount needed for chemically oxidizing organic and inorganic matter in water. COD is commonly used to assess the health of aquatic systems and to evaluate the effects of anthropogenic pressures, since elevated values often indicate industrial discharges, agricultural runoff, or urban effluents.

COD levels accurately forecasting is necessary for sustainable management of water quality and early mitigation of pollution events. However, COD cannot be regarded as a purely standalone response variable; its behavior is strongly influenced by hydrological processes such as flow regime, dilution effects, and seasonal variability, which alter concentration patterns and the underlying pollutant transport mechanisms. Neglecting these hydrological controls may oversimplify COD dynamics by assuming that they are driven solely by physicochemical variables, whereas in reality, COD variability emerges from the combined interaction of chemical, physical, and flow-related processes. This interdependence introduces additional nonlinearity and non-stationarity, posing a further modeling challenge for accurate COD prediction.

Traditional physically based models, though valuable for understanding processes, rely heavily on empirical equations and long-term datasets for calibration, which limits their transferability^1^. Statistical approaches, while faster and more economical, often assume linearity and normality, limiting their ability to capture the nonlinear relationships often found in hydrological systems^2^.

Recently, data-driven models have emerged as effective alternatives to statistical and physical approaches. Machine learning (ML) and deep learning (DL) models can learn complex nonlinear relationships directly from data without requiring explicit physical formulations^3^. These models offer adaptability and high predictive accuracy, making them suitable for dynamic environments where variable data quality and spatial heterogeneity are common^4–6^.

However, the performance of ML frameworks depends strongly on data scale, temporal resolution, and system characteristics, which often limit the direct comparability of published studies. For instance^7^, applied a multilayer perceptron (MLP) to predict COD in urban rivers of Wuxi, China, where nutrient loads were dominant, while^8^ used a Long Short-Term Memory–Recurrent Neural Network (LSTM-RNN) in a highly dynamic urban river network in Shanghai, emphasizing temporal continuity in COD variation^9^ demonstrated an edge artificial intelligence (edge-AI) approach for real-time, low-cost monitoring with a minimal sensor suite, including potential of hydrogen (pH), dissolved oxygen (DO), and BOD, whereas^10^ employed a Bidirectional Long Short-Term Memory (Bi-LSTM) model to address data gaps and irregular sampling in the Yamuna River. More recently^11^, combined neuro-fuzzy algorithms with metaheuristics to estimate COD in the same river, demonstrating the value of hybrid AI frameworks for complex water quality prediction. These applications differ not only in data availability but also in hydrological regimes, pollutant sources, and model objectives, highlighting that predictive strength alone does not ensure methodological transferability.

A rigorous conceptualization of ML frameworks has recently emerged in studies on horizontal flow constructed wetlands (HFCWs), where learning algorithms are explicitly linked to system design and process understanding^12^ used Support Vector Regression (SVR) and Multiple Linear Regression (MLR) to predict effluent BOD, COD, and nutrient levels from secondary datasets, showing that classifying data by organic loading rate improved interpretability and reduced error by 68%. Extending this approach^13^, combined ML with grey wolf optimization to identify optimal media depth for pollutant removal, integrating data-driven and mechanistic design principles^14^ further refined the first-order P–k–C* model by predicting realistic nutrient removal rate coefficients (k-values) through Artificial Neural Network (ANN), Random Forest (RF), and SVR models, while^15^ applied ML regionally to optimize wetland area requirements without compromising removal efficiency. Collectively, these studies show that ML can move beyond black-box prediction toward process-based understanding and design optimization, serving as a scientifically grounded complement to conventional physical models.

Building upon these advances, the present study investigates six boosting-based ensemble models—Adaptive Boosting (AdaBoost), Categorical Boosting (CatBoost), Extreme Gradient Boosting (XGBoost), Histogram Gradient Boosting (HistGBRT), Light Gradient Boosting (LightGBM), and Natural Gradient Boosting (NGBoost)—for COD prediction at two monitoring stations in South Korea. To enhance interpretability, the SHapley Additive exPlanations (SHAP) approach was employed to identify the contribution and influence of each input parameter on model outcomes^16^.

While previous research has examined COD or general water quality prediction using ML in Korea^17,18^, these studies have largely emphasized single-model prediction accuracy without addressing model interpretability or uncertainty. The novelty of this study lies in:

(i) the integration of multiple boosting-based ensemble models into a unified, explainable framework;
(ii) the comparative assessment of probabilistic (NGBoost) and deterministic boosting algorithms under identical hydro-environmental conditions, and
(iii) the use of SHAP-based global and local explanations to uncover the physical and chemical drivers of COD dynamics.

This hybrid evaluation–interpretation framework moves beyond conventional model benchmarking by linking predictive accuracy with process understanding and uncertainty quantification. Therefore, the study not only identifies the most reliable algorithm for COD forecasting in complex river systems but also provides an interpretable and transferable modeling strategy to support transparent, data-informed water quality management.

## Materials and methods

### Study area and available data

This study utilized various water quality and discharge parameters to predict COD concentrations at the Toilchun and Hwangji stations in South Korea. Since these two stations are located upstream of and close to the Yeongju Dam, a multipurpose dam in the region, their water quality and discharge characteristics can significantly influence eutrophication processes within the dam reservoir. Therefore, accurate prediction of COD at both stations can provide reliable data for assessing water quality within the reservoir boundary.

For both stations, long-term water quality datasets were available. As described earlier, evaluating different boosting-based ensemble models (i.e., AdaBoost, CatBoost, HistGBRT, LightGBM, NGBoost, and XGBoost) for COD prediction constitutes a central objective of this study. Another key aim is to extract information on how different models utilize the input parameters to make predictions.

Table 1 provides summary statistics of water quality and discharge indicators (i.e., pH, DO, BOD_5_, suspended solids (SS), total phosphorus (TP), total nitrogen (TN), total organic carbon (TOC), electrical conductivity (SC), water temperature (Tw), and station discharge (DIS)) at Toilchun Station. In the table, X_mean_ = average value; X_max_ = maximum value; X_min_ = minimum value; SD = the value of standard deviation; Cv = the value of variation coefficient; and R = the correlation coefficient between each input indicator and COD concentration. Table 1 shows that the standard deviation of the indicator SC is higher than that of the other indicators. Also, the indicators of SS and DIS exhibit higher Cv values than those of other indicators. The value of the R between each indicator and COD is also provided in Table 1 at Toilchun Station. The SS, TN, TP, TOC, Tw, DIS, and BOD_5_ exhibit positive R for corresponding COD concentrations, while the pH, DO, SC showed negative R for the corresponding COD concentration at Toilchun Station.

**Table 1:** Brief statistics for water quality variables at *Toilchun* station.

Variables	Subset	Unit	*X*_*mean*_	*X*_*max*_	*X*_*min*_	*SD*	*C*_*v*_	*R*
*pH*	Training	*/*	7.822	8.900	7.000	0.372	0.048	− 0.155
	Validation	*/*	7.817	9.000	7.100	0.323	0.041	− 0.092
	All data	*/*	7.820	9.000	7.000	0.358	0.046	− 0.134
*DO*	Training	*mg/L*	10.842	16.100	6.000	2.065	0.191	− 0.370
	Validation	*mg/L*	10.683	14.800	6.900	2.160	0.202	− 0.370
	All data	*mg/L*	10.794	16.100	6.000	2.093	0.194	− 0.370
*SS*	Training	*mg/L*	7.004	225.300	0.200	19.034	2.718	0.646
	Validation	*mg/L*	11.279	152.000	0.200	26.679	2.365	0.855
	All data	*mg/L*	8.282	225.300	0.200	21.655	2.615	0.732
*TN*	Training	*mg/L*	4.338	7.566	0.879	1.391	0.321	0.094
	Validation	*mg/L*	4.214	9.342	0.758	1.494	0.354	0.011
	All data	*mg/L*	4.301	9.342	0.758	1.422	0.331	0.062
*TP*	Training	*mg/L*	0.052	0.302	0.008	0.048	0.916	0.829
	Validation	*mg/L*	0.056	0.268	0.011	0.052	0.929	0.825
	All data	*mg/L*	0.054	0.302	0.008	0.049	0.920	0.827
*TOC*	Training	*mg/L*	2.483	10.300	0.500	1.290	0.520	0.930
	Validation	*mg/L*	2.697	10.900	0.600	1.589	0.589	0.966
	All data	*mg/L*	2.547	10.900	0.500	1.387	0.545	0.944
*Tw*	Training	*°C*	15.543	29.900	2.600	7.095	0.456	0.357
	Validation	*°C*	15.656	29.600	2.100	7.448	0.476	0.310
	All data	*°C*	15.577	29.900	2.100	7.192	0.462	0.340
*SC*	Training	*µ.s/cm*	272.535	355.000	129.000	42.893	0.157	− 0.413
	Validation	*µ.s/cm*	270.764	370.000	133.000	40.764	0.151	− 0.386
	All data	*µ.s/cm*	272.005	370.000	129.000	42.220	0.155	− 0.402
*DIS*	Training	*m*^*3/*^*s*	1.174	58.597	0.019	4.584	3.905	0.657
	Validation	*m*^*3/*^*s*	1.050	24.863	0.038	3.102	2.954	0.656
	All data	*m*^*3/*^*s*	1.137	58.597	0.019	4.192	3.688	0.634
*BOD*_*5*_	Training	*mg/L*	0.758	3.900	0.300	0.487	0.642	0.566
	Validation	*mg/L*	0.843	3.000	0.300	0.603	0.716	0.717
	All data	*mg/L*	0.783	3.900	0.300	0.525	0.670	0.626
*COD*	Training	*mg/L*	3.687	13.000	1.600	1.625	0.441	1.000
	Validation	*mg/L*	3.946	13.500	1.500	1.947	0.493	1.000
	All data	*mg/L*	3.764	13.500	1.500	1.729	0.459	1.000

**Fig. 1:**
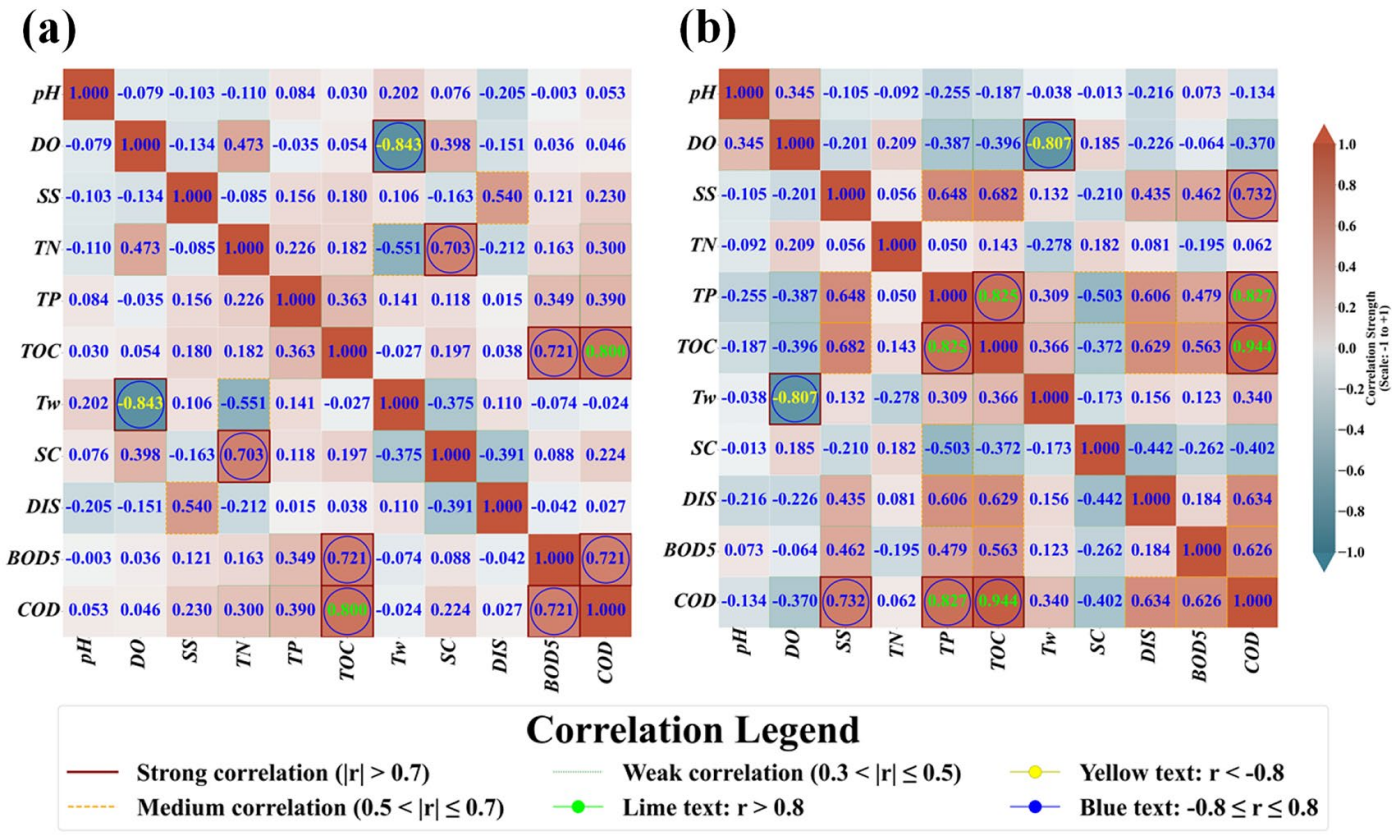
Heatmap correlation plot between water quality variables: (**a**) Hwangji station and (**b**) Toilchun station.

Table 2 supplies the summary statistics of water quality and discharge indicators including pH, DO, SS, TN, TP, TOC, Tw, SC, DIS, and BOD_5_ at Hwangji station. As shown in Table 2, the SD for the SC indicator was higher than that of the other indicators. Also, the indicators of SS and DIS provided higher variation coefficients than those of other indicators. The value of the R between each indicator and COD was also given in Table 2 at Hwangji station. The indicators of pH, DO, SS, TN, TP, TOC, SC, DIS, and BOD_5_ produced a positive R for the corresponding COD, whereas the Tw indicator gave a negative R for the corresponding COD at Toilchun Station. Figure 1 illustrates the heatmap correlation between the water quality variables at the two monitoring stations.

**Table 2:** Summary statistics of water quality variables at *Hwangji* station.

Variables	Subset	Unit	*X*_*mean*_	*X*_*max*_	*X*_*min*_	*SD*	*C*_*v*_	*R*
*pH*	Training	*/*	8.216	9.100	7.000	0.355	0.043	0.041
	Validation	*/*	8.249	9.000	7.000	0.348	0.042	0.077
	All data	*/*	8.226	9.100	7.000	0.353	0.043	0.053
*DO*	Training	*mg/L*	11.376	17.200	7.800	2.055	0.181	0.042
	Validation	*mg/L*	11.839	17.500	7.300	2.224	0.188	0.044
	All data	*mg/L*	11.515	17.500	7.300	2.116	0.184	0.046
*SS*	Training	*mg/L*	3.659	48.000	0.200	5.841	1.597	0.532
	Validation	*mg/L*	7.285	266.000	0.300	26.968	3.702	0.168
	All data	*mg/L*	4.746	266.000	0.200	15.609	3.289	0.230
*TN*	Training	*mg/L*	3.363	7.215	1.824	0.841	0.250	0.277
	Validation	*mg/L*	3.487	7.559	1.726	1.025	0.294	0.348
	All data	*mg/L*	3.400	7.559	1.726	0.901	0.265	0.300
*TP*	Training	*mg/L*	0.042	0.151	0.005	0.029	0.685	0.347
	Validation	*mg/L*	0.051	0.218	0.004	0.034	0.677	0.476
	All data	*mg/L*	0.045	0.218	0.004	0.031	0.689	0.390
*TOC*	Training	*mg/L*	1.937	9.400	0.800	0.788	0.407	0.793
	Validation	*mg/L*	2.022	5.600	1.000	0.668	0.330	0.823
	All data	*mg/L*	1.963	9.400	0.800	0.754	0.384	0.800
*Tw*	Training	*°C*	12.914	26.300	0.000	6.890	0.534	− 0.017
	Validation	*°C*	12.145	25.000	− 1.000	6.942	0.572	− 0.033
	All data	*°C*	12.683	26.300	− 1.000	6.907	0.545	− 0.024
*SC*	Training	*µ.s/cm*	456.948	794.000	201.000	120.582	0.264	0.210
	Validation	*µ.s/cm*	461.362	823.000	191.000	130.089	0.282	0.256
	All data	*µ.s/cm*	458.272	823.000	191.000	123.393	0.269	0.224
*DIS*	Training	*m*^*3/*^*s*	5.094	74.903	0.488	9.630	1.891	0.044
	Validation	*m*^*3/*^*s*	7.282	330.650	0.630	28.750	3.948	0.015
	All data	*m*^*3/*^*s*	5.750	330.650	0.488	17.678	3.075	0.027
*BOD*_*5*_	Training	*mg/L*	1.157	9.000	0.300	0.852	0.737	0.736
	Validation	*mg/L*	1.246	6.500	0.300	0.739	0.593	0.680
	All data	*mg/L*	1.184	9.000	0.300	0.820	0.693	0.721
*COD*	Training	*mg/L*	3.216	10.000	1.600	0.960	0.298	1.000
	Validation	*mg/L*	3.301	7.300	1.500	0.927	0.281	1.000
	All data	*mg/L*	3.241	10.000	1.500	0.950	0.293	1.000

It is noted that some correlation coefficients are similar between both stations (e.g. the negative correlation between Tw and DO), while other correlation coefficients differ between stations (e.g. SC versus COD). There are common model characteristics, representing underlying general correlations, and regional or specific characteristics, pertaining to the different basins.

Also, various arrangements of water quality and discharge indicators were chosen to build different input combinations. Therefore, six boosting ensemble models were made for predicting COD concentration depending on nine input combinations of different complexity (Table 3). Since the indicators of TOC and SC were selected as the basic unit for building input combinations, this research used the indicators of TOC and SC as the 9th input combination to predict COD concentration. In addition, the first input combination consists of all ten available indicators including pH, DO, SS, TN, TP, TOC, Tw, SC, DIS, and BOD_5_. The seven intermediate input combinations were obtained by gradually reducing the number of parameters and model complexity, omitting pH, DO, Tw, Dis, Tp, BOD, SS and TN.

**Table 3:** The input combinations of the Boosting models.

Models						Inputs	Output
AdaBoost1	CatBoost1	HistGBRT1	LightGBM1	NGBoost1	XGBoost1	pH, DO, SS, TN, TP, TOC, Tw, SC, DIS, BOD_5_	COD
AdaBoost2	CatBoost2	HistGBRT2	LightGBM2	NGBoost2	XGBoost2	DO, SS, TN, TP, TOC, Tw, SC, DIS, BOD_5_	COD
AdaBoost3	CatBoost3	HistGBRT3	LightGBM3	NGBoost3	XGBoost3	SS, TN, TP, TOC, Tw, SC, DIS, BOD_5_	COD
AdaBoost4	CatBoost4	HistGBRT4	LightGBM4	NGBoost4	XGBoost4	SS, TN, TP, TOC, SC, DIS, BOD_5_	COD
AdaBoost5	CatBoost5	HistGBRT5	LightGBM5	NGBoost5	XGBoost5	SS, TN, TP, TOC, SC, BOD_5_	COD
AdaBoost6	CatBoost6	HistGBRT6	LightGBM6	NGBoost6	XGBoost6	SS, TN, TOC, SC, BOD_5_	COD
AdaBoost7	CatBoost7	HistGBRT7	LightGBM7	NGBoost7	XGBoost7	SS, TN, TOC, SC	COD
AdaBoost8	CatBoost8	HistGBRT8	LightGBM8	NGBoost8	XGBoost8	TN, TOC, SC	COD
AdaBoost9	CatBoost9	HistGBRT9	LightGBM9	NGBoost9	XGBoost9	TOC, SC	COD

**Fig. 2:**
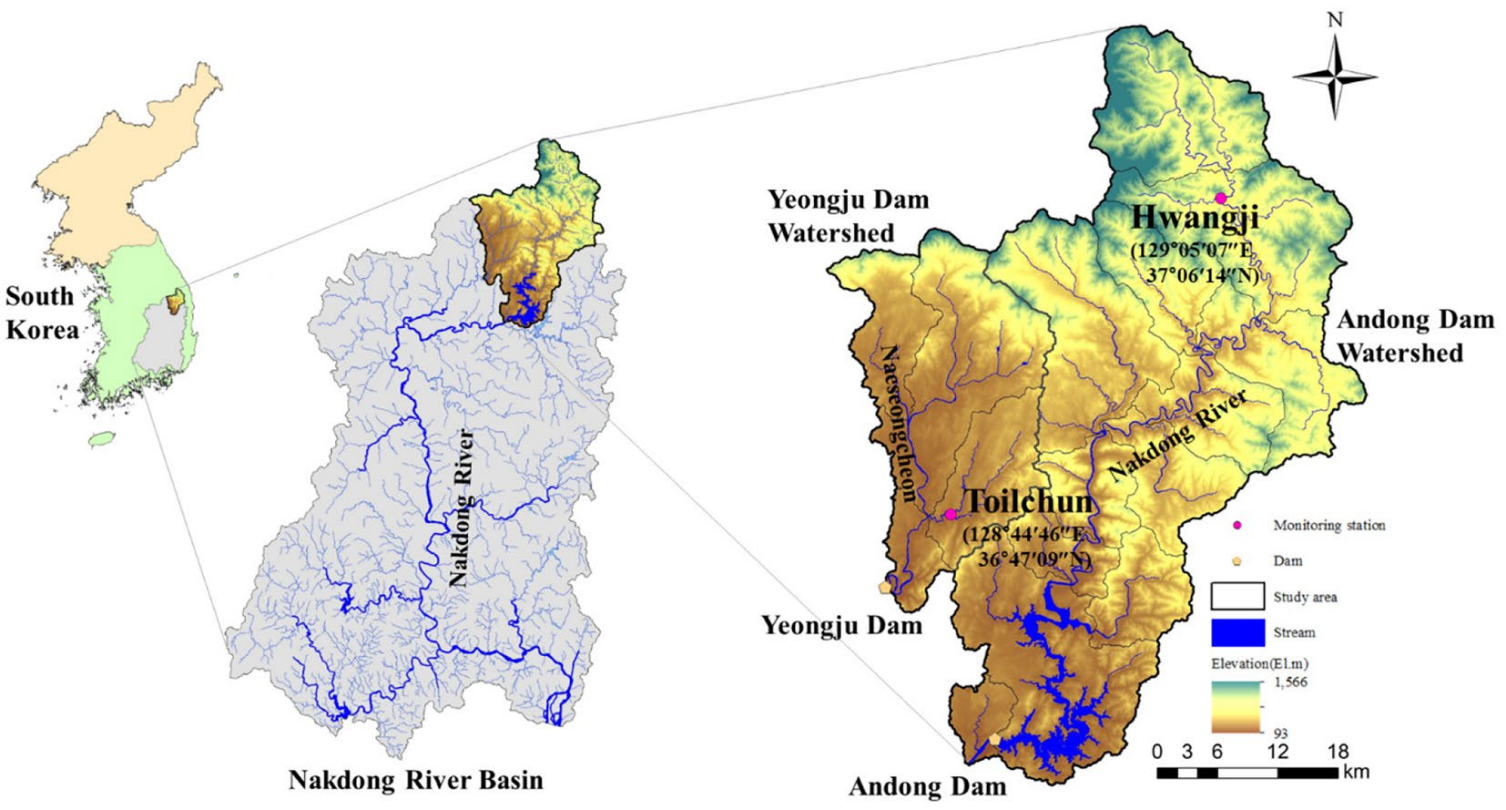
Schematic map of water quality and discharge stations, South Korea.

Figure 2 presents the schematic map of water quality and discharge stations, South Korea. The dataset available for water quality and discharge can be directly accessed and downloaded from the website (http://water.nier.go.kr). The period of the collected dataset corresponds to the period from 2011/07–2020/12 for Toilchun (longitude 128°44′46″E; latitude 36°47′09″N) and 2008/02–2020/12 for Hwangji (longitude 129°05′07″E; latitude 37°06′74″N) stations, respectively. In addition, the training length of the collected dataset involved the first 70% (i.e., n = 258 for Toilchun and n = 348 for Hwangji stations), and the validation length involved the remaining 30% (i.e., n = 110 for Toilchun and n = 149 for Hwangji stations) of the collected dataset. The respective statistics of the training, validation, and entire datasets were calculated and provided in Tables 1 and 2 for both stations as underlying information regarding the similarity of training and validation records.

### Accuracy evaluation criteria

Root-mean-square error (RMSE), mean absolute error (MAE), Nash–Sutcliffe efficiency (NSE), correlation coefficient (R), and Percent Bias (PBIAS) were used for assessing models’ accuracy.

1 $$MAE=\frac{\sum_{i=1}^{N}|{COD}_{pre,i}-{COD}_{obs,i}|}{N}$$

2 $$RMSE=\sqrt{\frac{\sum_{i=1}^{N}({COD}_{obs,i}- {COD}_{pre,i}{)}^{2}}{N}}$$

3 $$NSE=1-\left[\frac{\sum_{i=1}^{N}({COD}_{obs,i}- {COD}_{pre,i}{)}^{2}}{\sum_{i=1}^{N}({{COD}_{obs,i}- \overline{{COD }_{obs}})}^{2}}\right]$$

4 $$R=\left(\frac{\sum_{i=1}^{N}\left({COD}_{obs,i}- \overline{{COD }_{obs}}\right) \left({COD}_{pre,i}- \overline{{COD }_{pre}}\right) }{\sqrt{\sum_{i=1}^{N}({{COD}_{obs,i}- \overline{{COD }_{obs}})}^{2} \sum_{i=1}^{N}({{COD}_{pre,i}- \overline{{COD }_{pre}})}^{2} }}\right)$$

5 $$PBIAS=\frac{{\sum }_{i}^{N}\left({COD}_{pre,i}-{COD}_{obs,i}\right)}{{\sum }_{i}^{N}{COD}_{obs,i}}\times 100$$

Here ${COD}_{pre,i}$ and ${COD}_{obs,i}$ represent calculated and measured daily *COD* at an *ith* time step, *N* is the observation quantity, $\overline{{COD }_{pre}}$ and $\overline{{COD }_{obs}}$ are the means of calculated and measured daily *COD*.

## Machine learning models

### AdaBoost (Adaptive boosting)

The AdaBoost algorithm, developed by^19^, is an ensemble learning method that combines multiple weak classifiers to form a strong predictive model. In AdaBoost, bias control is achieved by integrating several boosting iterations into a consolidated prediction framework. During training, data samples misclassified by previous classifiers are assigned higher weights and reused to train subsequent classifiers^20^. Compared with other boosting-based ensemble methods, AdaBoost is generally less prone to overfitting or underfitting in specific classification problems. The base classifier used in AdaBoost may be weak; that is, it initially exhibits relatively high classification error but performs better than random guessing. The overall predictive performance of AdaBoost improves through a weighted voting process, where the final decision depends on the combined output of all weak classifiers^21,22^. AdaBoost operates iteratively, updating the weak classifier at each step until the overall classification error converges to a minimum. The weight assigned to each sample is adjusted based on its classification accuracy: correctly classified samples receive lower weights, while misclassified samples receive higher weights, thereby increasing their influence in the next iteration^19,23^. Through this adaptive weighting mechanism, AdaBoost enhances the precision of weak learners by focusing on more challenging samples. Ultimately, the collection of weak classifiers is integrated to construct a robust composite model^19,24^. The algorithm thus improves predictive accuracy by emphasizing samples with higher training errors and iteratively refining the ensemble with stronger classifiers^22,23^. Detailed formulations and applications of the AdaBoost algorithm can be found in^19,22,23^.

### CatBoost (Categorical boosting)

The CatBoost algorithm, developed by^25^, provides a practical and precise approach for handling categorical features during model training. It enhances generalization performance by addressing issues related to bias and variance, thereby improving prediction accuracy and model stability. To reduce the risk of overfitting or underfitting, CatBoost employs an advanced technique known as ordered boosting, a refined version of traditional gradient boosting that enhances learning efficiency and model generalization^26–28^. In the CatBoost framework, training samples are arranged according to a predefined order to construct multiple models based on sequential training subsets^29^. This ordered sampling strategy prevents target leakage by ensuring that each model is trained only on data available before the prediction target, thus maintaining unbiased learning. CatBoost also employs target-based encoding to convert categorical variables into numerical representations. For each observation, numerical transformations are computed using target statistics derived from prior training data, allowing the model to assign appropriate weights and priorities to categorical features^25,30^. Furthermore, CatBoost adopts a greedy, iterative optimization process to minimize a specified loss function efficiently^25,31^. This combination of ordered boosting, target encoding, and iterative optimization enables CatBoost to achieve superior predictive performance while maintaining robustness against overfitting. Detailed explanations and applications of the CatBoost algorithm for various predictive modeling tasks can be found in^25,27^.

### HistGBRT (Histogram gradient boosting)

The HistGBRT model is a modified version of the well-known gradient boosting, which is commonly used for solving various gradient boosting problems in classification and regression issues^32^. The primary objective of HistGBRT is to transform weak learners into a strong predictive model by sequentially adding new weak classifiers that correct the residual errors of previously fitted ones. In other words, each weak learner is trained to reduce the mistakes made by the preceding classifiers^33–35^. HistGBRT was developed to overcome one of the main drawbacks of traditional gradient boosting, its long training time when applied to large datasets. This limitation is addressed by discretizing continuous input features into a fixed number of discrete bins, thereby constructing histograms that approximate the feature distributions. This process substantially reduces computational complexity and accelerates training. Among the hyperparameters of the HistGBRT model, the learning rate plays a particularly important role in balancing model accuracy and overfitting^36^. Unlike standard gradient boosting approaches, HistGBRT stores continuous feature values in separate containers (bins) and utilizes these binned values to build histograms during model training. This histogram-based approach not only accelerates computation but also significantly reduces memory usage, making HistGBRT well-suited for large-scale data analysis^32,37^. Comprehensive discussions and applications demonstrating the performance of the HistGBRT model can be found in^32,36,37^.

### LightGBM (Light gradient boosting machine)

The LightGBM algorithm is an advanced form of gradient boosting, developed to address computational challenges associated with earlier boosting techniques, such as long training times, high complexity, and memory inefficiency. It uses decision trees as base learners within a gradient boosting framework, enabling the model to handle both regression and classification problems effectively^38,39^. To improve computational efficiency and model performance, LightGBM introduces two key innovations: Gradient-based One-Side Sampling (GOSS) and Exclusive Feature Bundling (EFB). These techniques significantly reduce computational cost while maintaining high prediction accuracy^40,41^. The LightGBM model employs the histogram algorithm combined with a depth-limiting strategy for leaf-wise growth to reduce memory loss. It also achieves higher accuracy with lower computational overhead while mitigating the risks associated with the gradient boosting decision tree (GBDT) model^42,43^. Furthermore, LightGBM incorporates a maximum depth constraint and other structural design features to further enhance efficiency and model stability during training^40,41^. Another advantage of LightGBM is its ability to handle categorical variables directly. Unlike many machine learning algorithms that require one-hot encoding for categorical inputs, a process that often increases dimensionality and reduces efficiency, LightGBM processes categorical features natively, thereby improving both computational speed and predictive accuracy^43,44^. Comprehensive discussions and applications of the LightGBM algorithm for various predictive modeling tasks can be found in^40,41,43,44^.

### NGBoost (Natural gradient boosting)

NGBoost model, which employs natural gradient boosting, combines scoring rules, types of probability distribution, and base learners^45–47^. Conventional gradient boosting models build predictive systems by modeling correlations between inputs and outputs^47^. This method creates a comprehensive, probabilistic model for input and output parameters, enabling the calculation of predictive uncertainty via probabilistic prediction^38,48^. In addition, the NGBoost model incorporates natural gradients with ensemble methods. By organizing individual models that adjust to natural gradients, it builds a compound predictor within the gradient boosting scheme. It allows the system to calculate the distribution parameters, eventually assisting probability prediction^47–49^. The NGBoost model can enhance the original one. For estimating multiple parameters, the original model, based on gradient boosting and employing a single parameter, may be suboptimal with respect to the gradients of other parameters^48^. The natural gradient can be increased by the inverse method of the Riemann metric to adjust multiple parameters against conventional gradient, also admitting it to carry out boosting of multiple parameters^38,48^. Unlike the other gradient boosting variants used in this study which provide deterministic point estimates, NGBoost’s probabilistic framework allows it to model the entire conditional distribution of the target variable. This special feature of the NGBoost technique is critical because it enables the model to account for predictive uncertainty. Also, this characteristic can significantly enhance generalization on unseen validation data. The featured instruction for the development and investigation of the NGBoost model to address predictive issues can be expressed from various documents, including^45–48^.

### XGBoost (Extreme gradient boosting)

XGBoost, an outstanding model employed for supervised training, was introduced by^50^. It is an approach that assumes a gradient boosting classifier and has a significant impact on gradient enhancement^51,52^. It serves as a powerful solution for regression and classification problems by applying the classifier theorem to them^53^. The XGBoost model is built on the concept of boosting^54^. In practice, boosting combines several simple models, and each of them makes predictions only slightly better than a random selection model. By combining these models into an ensemble that performs slightly better than a random model via boosting, developers and modelers can create a single powerful model that makes highly accurate predictions^55^. In predictive modeling, ensemble learning combines multiple weak learners to produce a stronger and more accurate predictive system. Each base learner contributes a partial estimation of the target variable, and their aggregated results help minimize bias and variance^50,56^. XGBoost applies this principle by constructing an ensemble of Classification and Regression Trees (CARTs), where each successive tree focuses on correcting the residuals of the previous ones. Through iterative boosting and aggregation, XGBoost achieves high flexibility and robustness, making it suitable for a wide range of classification and regression tasks^50^. Comprehensive explanations and applications of the XGBoost algorithm can be found in^50,52,53^.

## Results and discussion

**Fig. 3:**
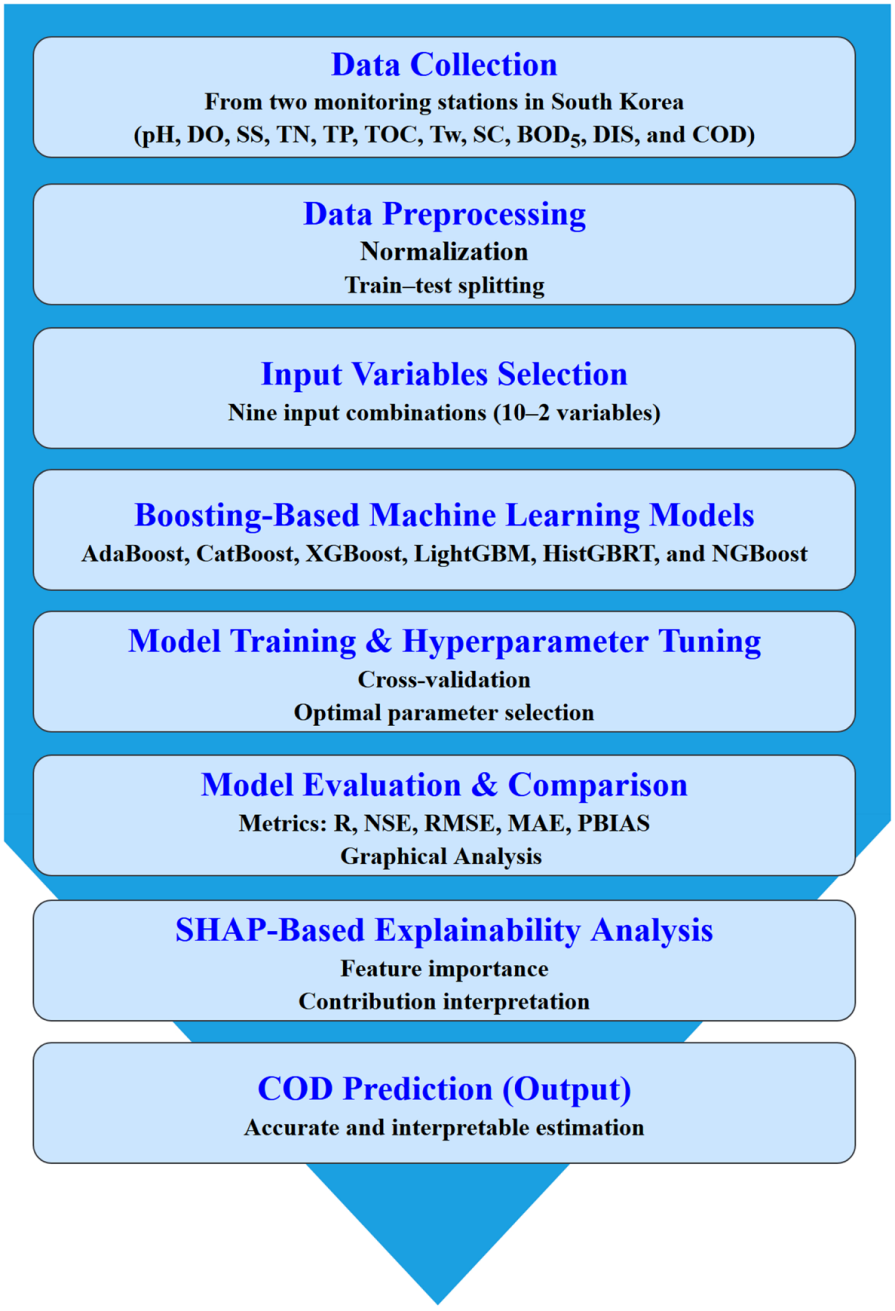
Flowchart summarizing the overall study steps.

This section presents the representation and analysis of predicted COD values using six boosting models: AdaBoost, CatBoost, XGBoost, HistGBRT, LightGBM, and NGBoost. The results are discussed in three distinct parts: *(i)* mathematical analysis based on evaluation criteria, *(ii)* diagrammatic analysis through visualization, and *(iii)* interpretation using SHAP via bar plots, beeswarm plots, force plots, and waterfall plots. Finally, a general discussion of the study findings is provided. Figure 3 illustrates the overall study flowchart.

### Mathematical analysis based on the evaluation criteria

The analysis of the results for predicting COD using six ML models at the two stations is presented in Tables 4 and 5 for the Hwangji and Toilchun Stations, respectively. In these tables, the best model for each boosting category is highlighted. As can be concluded from the statistical measures for the training and validation sets across both stations, model accuracy generally decreases (lower values for the R and NSE and higher values for the RMSE and MAE) when moving from the training set (70% of the total data) to the validation set (30% of the total data). This trend is typical, as models tend to fit the training data better than unseen validation data. For the Hwangji Station (Table 4), NGBoost and CatBoost show more stable results on the validation set than the other boosting methods (with NGBoost5 achieving an R value of about 0.842 and CatBoost1 around 0.861). While XGBoost5 performs well on the training set, it shows higher RMSE (= 0.588 mg/L) and MAE (= 0.435 mg/L) on the validation set. This matter might indicate potential overfitting at this station. From a scientific perspective, the overfitting observed in models like XGBoost, characterized by near-perfect training metrics but a significant performance drop in validation, has critical implications for environmental inference. Overfitting suggests that the model is learning specific noise or transient anomalies in the water quality data rather than the robust biochemical relationships governing COD. Consequently, such models may provide misleading results when used for real-time monitoring or policy-making. This highlights the necessity of using more stable ensemble methods, such as NGBoost and CatBoost, which demonstrate better generalization and thus offer more reliable scientific insights into pollution dynamics. This systematic performance gap confirms the inherent risks of over-parameterization in environmental datasets. When models overfit, the resulting feature importance rankings (e.g., via SHAP) may be biased toward variables that explain local variance in the training set rather than the actual drivers of organic pollution. On the other hand, the CatBoost1 model provides more consistent prediction errors in comparison to the other applied models and can be introduced as the most accurate model.

**Table 4:** Performances of different boosting models for COD prediction at *Hwangji* station.

Model	Training					Validation				
	R	NSE	RMSE	MAE	PBIAS	R	NSE	RMSE	MAE	PBIAS
			(mg/L)	(mg/L)	(%)			(mg/L)	(mg/L)	(%)
AdaBoost1	0.907	0.819	0.408	0.337	− 0.368	0.807	0.642	0.553	0.401	− 0.171
AdaBoost2	0.907	0.819	0.408	0.337	0.408	0.811	0.649	0.547	0.394	0.385
AdaBoost3	0.906	0.818	0.409	0.340	0.329	0.803	0.641	0.554	0.399	0.375
AdaBoost4*	0.903	0.814	0.413	0.343	0.469	0.816	0.660	0.539	0.392	0.518
AdaBoost5	0.900	0.807	0.420	0.351	0.838	0.805	0.643	0.552	0.395	0.521
AdaBoost6	0.896	0.800	0.429	0.361	0.905	0.804	0.641	0.554	0.399	0.397
AdaBoost7	0.873	0.760	0.470	0.388	0.661	0.743	0.547	0.622	0.478	1.353
AdaBoost8	0.835	0.695	0.530	0.440	1.266	0.765	0.575	0.603	0.479	1.237
AdaBoost9	0.819	0.670	0.551	0.441	0.233	0.772	0.558	0.615	0.474	− 1.029
CatBoost1*	0.884	0.773	0.456	0.355	− 0.368	0.861	0.733	0.477	0.364	− 0.171
CatBoost2	0.888	0.777	0.452	0.350	0.408	0.853	0.709	0.498	0.376	0.385
CatBoost3	0.884	0.775	0.455	0.351	0.329	0.849	0.709	0.499	0.377	0.375
CatBoost4	0.888	0.779	0.451	0.353	0.469	0.838	0.691	0.514	0.385	0.518
CatBoost5	0.885	0.774	0.455	0.349	0.838	0.857	0.721	0.488	0.362	0.521
CatBoost6	0.879	0.767	0.463	0.360	0.905	0.854	0.720	0.489	0.385	0.397
CatBoost7	0.855	0.724	0.504	0.394	0.661	0.813	0.656	0.542	0.428	1.353
CatBoost8	0.829	0.683	0.540	0.425	1.266	0.815	0.659	0.539	0.435	1.237
CatBoost9	0.818	0.666	0.554	0.427	0.233	0.818	0.659	0.540	0.438	− 1.029
HistGBRT1	0.951	0.898	0.307	0.163	− 0.368	0.819	0.667	0.533	0.400	− 0.171
HistGBRT2	0.949	0.895	0.310	0.168	0.408	0.813	0.658	0.540	0.410	0.385
HistGBRT3	0.949	0.894	0.311	0.175	0.329	0.809	0.652	0.545	0.419	0.375
HistGBRT4	0.944	0.885	0.325	0.188	0.469	0.807	0.646	0.550	0.424	0.518
HistGBRT5*	0.932	0.863	0.354	0.204	0.838	0.828	0.679	0.523	0.408	0.521
HistGBRT6	0.915	0.833	0.391	0.232	0.905	0.829	0.678	0.525	0.412	0.397
HistGBRT7	0.910	0.821	0.405	0.252	0.661	0.759	0.557	0.615	0.493	1.353
HistGBRT8	0.875	0.758	0.472	0.307	1.266	0.751	0.564	0.610	0.476	1.237
HistGBRT9	0.808	0.648	0.569	0.378	0.233	0.743	0.550	0.620	0.473	− 1.029
LightGBM1	0.949	0.894	0.311	0.169	− 0.368	0.819	0.668	0.532	0.401	− 0.171
LightGBM2	0.950	0.895	0.311	0.173	0.408	0.814	0.661	0.538	0.414	0.385
LightGBM3	0.947	0.890	0.317	0.179	0.329	0.818	0.665	0.534	0.405	0.375
LightGBM4	0.943	0.882	0.330	0.195	0.469	0.810	0.652	0.545	0.415	0.518
LightGBM5*	0.927	0.854	0.366	0.212	0.838	0.833	0.688	0.516	0.403	0.521
LightGBM6	0.915	0.832	0.393	0.237	0.905	0.831	0.681	0.522	0.408	0.397
LightGBM7	0.907	0.816	0.411	0.260	0.661	0.767	0.569	0.606	0.490	1.353
LightGBM8	0.870	0.748	0.481	0.316	1.266	0.750	0.562	0.612	0.475	1.237
LightGBM9	0.800	0.636	0.578	0.383	0.233	0.738	0.543	0.624	0.474	− 1.029
NGBoost1	0.963	0.922	0.267	0.213	− 0.368	0.837	0.698	0.508	0.375	− 0.171
NGBoost2	0.961	0.920	0.271	0.216	0.408	0.835	0.694	0.511	0.380	0.385
NGBoost3	0.961	0.920	0.272	0.216	0.329	0.839	0.701	0.505	0.375	0.375
NGBoost4	0.958	0.914	0.281	0.224	0.469	0.831	0.689	0.516	0.385	0.518
NGBoost5*	0.955	0.908	0.291	0.231	0.838	0.842	0.705	0.502	0.372	0.521
NGBoost6	0.949	0.897	0.307	0.246	0.905	0.832	0.689	0.515	0.386	0.397
NGBoost7	0.927	0.855	0.365	0.290	0.661	0.787	0.606	0.580	0.439	1.353
NGBoost8	0.908	0.819	0.408	0.321	1.266	0.815	0.660	0.539	0.431	1.237
NGBoost9	0.898	0.804	0.425	0.332	0.233	0.818	0.666	0.534	0.430	− 1.029
XGBoost1	0.999	0.999	0.001	0.001	− 0.368	0.773	0.595	0.588	0.437	− 0.171
XGBoost2	0.999	0.999	0.001	0.001	0.408	0.767	0.587	0.594	0.440	0.385
XGBoost3	0.999	0.999	0.003	0.002	0.329	0.748	0.559	0.614	0.444	0.375
XGBoost4	0.999	0.999	0.003	0.002	0.469	0.758	0.575	0.602	0.439	0.518
XGBoost5*	0.999	0.999	0.006	0.004	0.838	0.774	0.599	0.585	0.435	0.521
XGBoost6	0.999	0.999	0.006	0.004	0.905	0.762	0.571	0.605	0.452	0.397
XGBoost7	0.999	0.999	0.011	0.007	0.661	0.739	0.526	0.636	0.506	1.353
XGBoost8	0.999	0.999	0.032	0.023	1.266	0.732	0.530	0.633	0.523	1.237
XGBoost9	0.992	0.984	0.122	0.070	0.233	0.749	0.560	0.613	0.500	− 1.029

The best model for each category is highlighted by an asterisk (*).

**Table 5:** Performances of different models for COD prediction at *Toilchun* station.

Model	Training					Validation				
	R	NSE	RMSE	MAE	PBIAS	R	NSE	RMSE	MAE	PBIAS
			(mg/L)	(mg/L)	(%)			(mg/L)	(mg/L)	(%)
AdaBoost1	0.971	0.940	0.397	0.333	− 0.368	0.966	0.928	0.521	0.387	− 0.171
AdaBoost2	0.972	0.941	0.395	0.329	0.408	0.965	0.926	0.527	0.396	0.385
AdaBoost3	0.972	0.941	0.393	0.327	0.329	0.966	0.929	0.517	0.395	0.375
AdaBoost4	0.972	0.941	0.392	0.324	0.469	0.967	0.931	0.510	0.378	0.518
AdaBoost5	0.969	0.935	0.413	0.345	0.838	0.965	0.927	0.524	0.396	0.521
AdaBoost6	0.968	0.934	0.417	0.347	0.905	0.966	0.931	0.510	0.399	0.397
AdaBoost7*	0.964	0.928	0.436	0.360	0.661	0.968	0.934	0.499	0.380	1.353
AdaBoost8	0.958	0.917	0.468	0.372	1.266	0.965	0.926	0.527	0.394	1.237
AdaBoost9	0.955	0.910	0.485	0.387	0.233	0.963	0.925	0.531	0.414	− 1.029
CatBoost1*	0.970	0.940	0.397	0.317	− 0.368	0.974	0.948	0.442	0.336	− 0.171
CatBoost2	0.968	0.936	0.409	0.322	0.408	0.972	0.945	0.456	0.341	0.385
CatBoost3	0.968	0.937	0.408	0.318	0.329	0.970	0.940	0.475	0.352	0.375
CatBoost4	0.966	0.931	0.425	0.333	0.469	0.969	0.940	0.476	0.365	0.518
CatBoost5	0.961	0.922	0.453	0.350	0.838	0.971	0.943	0.464	0.358	0.521
CatBoost6	0.961	0.923	0.450	0.358	0.905	0.974	0.948	0.442	0.356	0.397
CatBoost7	0.956	0.913	0.478	0.370	0.661	0.972	0.945	0.456	0.355	1.353
CatBoost8	0.950	0.902	0.509	0.372	1.266	0.959	0.909	0.584	0.408	1.237
CatBoost9	0.948	0.899	0.515	0.385	0.233	0.970	0.934	0.497	0.365	− 1.029
HistGBRT1	0.973	0.945	0.380	0.193	− 0.368	0.942	0.887	0.653	0.441	− 0.171
HistGBRT2	0.971	0.941	0.394	0.202	0.408	0.944	0.890	0.642	0.425	0.385
HistGBRT3*	0.971	0.940	0.396	0.208	0.329	0.944	0.892	0.638	0.424	0.375
HistGBRT4	0.970	0.938	0.403	0.217	0.469	0.944	0.891	0.638	0.431	0.518
HistGBRT5	0.968	0.936	0.412	0.236	0.838	0.942	0.888	0.649	0.428	0.521
HistGBRT6	0.964	0.928	0.434	0.254	0.905	0.944	0.890	0.641	0.426	0.397
HistGBRT7	0.950	0.901	0.509	0.283	0.661	0.935	0.871	0.696	0.417	1.353
HistGBRT8	0.934	0.871	0.582	0.323	1.266	0.932	0.862	0.720	0.422	1.237
HistGBRT9	0.921	0.848	0.633	0.381	0.233	0.922	0.844	0.766	0.466	− 1.029
LightGBM1	0.971	0.941	0.393	0.204	− 0.368	0.940	0.883	0.662	0.436	− 0.171
LightGBM2	0.970	0.939	0.401	0.212	0.408	0.940	0.884	0.661	0.438	0.385
LightGBM3*	0.970	0.939	0.401	0.217	0.329	0.945	0.893	0.634	0.420	0.375
LightGBM4	0.967	0.934	0.416	0.227	0.469	0.945	0.892	0.637	0.428	0.518
LightGBM5	0.966	0.932	0.422	0.243	0.838	0.942	0.886	0.653	0.435	0.521
LightGBM6	0.963	0.926	0.441	0.260	0.905	0.944	0.892	0.638	0.427	0.397
LightGBM7	0.949	0.899	0.515	0.289	0.661	0.939	0.877	0.679	0.415	1.353
LightGBM8	0.934	0.872	0.580	0.325	1.266	0.934	0.864	0.714	0.426	1.237
LightGBM9	0.919	0.845	0.639	0.387	0.233	0.922	0.843	0.768	0.462	− 1.029
NGBoost1	0.994	0.987	0.183	0.147	− 0.368	0.973	0.946	0.450	0.329	− 0.171
NGBoost2	0.994	0.987	0.183	0.149	0.408	0.972	0.944	0.457	0.332	0.385
NGBoost3	0.994	0.987	0.184	0.150	0.329	0.973	0.946	0.449	0.325	0.375
NGBoost4	0.992	0.984	0.203	0.163	0.469	0.973	0.946	0.451	0.332	0.518
NGBoost5	0.991	0.981	0.225	0.177	0.838	0.973	0.947	0.448	0.333	0.521
NGBoost6	0.989	0.978	0.241	0.192	0.905	0.977	0.955	0.412	0.335	0.397
NGBoost7*	0.987	0.973	0.266	0.204	0.661	0.979	0.958	0.397	0.323	1.353
NGBoost8	0.985	0.970	0.282	0.220	1.266	0.975	0.948	0.441	0.349	1.237
NGBoost9	0.978	0.956	0.339	0.258	0.233	0.968	0.936	0.489	0.373	− 1.029
XGBoost1	0.999	0.999	0.001	0.001	− 0.368	0.964	0.928	0.521	0.371	− 0.171
XGBoost2	0.999	0.999	0.001	0.001	0.408	0.968	0.935	0.495	0.362	0.385
XGBoost3	0.999	0.999	0.001	0.001	0.329	0.964	0.926	0.529	0.380	0.375
XGBoost4	0.999	0.999	0.001	0.001	0.469	0.964	0.928	0.522	0.387	0.518
XGBoost5	0.999	0.999	0.001	0.001	0.838	0.966	0.932	0.506	0.388	0.521
XGBoost6	0.999	0.999	0.002	0.001	0.905	0.965	0.931	0.508	0.395	0.397
XGBoost7*	0.999	0.999	0.003	0.002	0.661	0.971	0.941	0.470	0.357	1.353
XGBoost8	0.999	0.999	0.012	0.007	1.266	0.956	0.913	0.571	0.435	1.237
XGBoost9	0.999	0.998	0.078	0.036	0.233	0.950	0.902	0.607	0.450	− 1.029

The best model for each category is highlighted by an asterisk (*).

At the Toilchun station (Table 5), the NGBoost and XGBoost models achieve the highest R values during training (NGBoosts 1, 2, 3 = 0.994, XGBoosts 1, 2, 7 = 1.000), but in total, the NGBoost maintains a more stable validation (R > 0.972 and NSE > 0.936) compared to XGBoost. This means that the NGBoost is more reliable for this specific application. The superior performance and stability of NGBoost can be scientifically attributed to its probabilistic prediction capability. While models like XGBoost and HistGBRT focus on minimizing a loss function based on deterministic residuals, NGBoost estimates the parameters of a probability distribution. This feature allows the model to better navigate the stochastic nature of water quality data. The statistic measures, including RMSE and MAE, also highlight NGBoosts lower RMSE values during validation (NGBoost7, RMSE = 0.470 mg/L and MAE = 0.357 mg/L), indicating more accurate predictions. In contrast, HistGBRT and LightGBM provide higher RMSE values, which indicate they are not as accurate as the other boosting models for the prediction of COD at this station. An interesting point is the different behavior of the applied models using various input parameters, as given in Table 3. For both stations, the Catboost1 model acts the best when it uses all of the 10 independent variables (pH, DO, SS, TN, TP, TOC, Tw, SC, DIS, BOD_5_), however, other models such as NGBoost7 and XGBoost7 act better in prediction using the seventh combination as inputs (SS, TN, TOC, SC). Comparing the provided results of the two stations, the overall performance of the models for the Hwangji Station is slightly lower compared to Toilchun Station, which may be due to differences in data characteristics, as seen in the summary statistics (Tables 2, 3). Although the NGBoost model does not provide the best error metrics in the training set, it was the most accurate model in predicting the COD values based on the error metrics in the validation set.

The systematic drop in validation performance across both stations confirms the generalization limits inherent in high-variance models like gradient boosting machines. While these models capture complex patterns in training data, their sensitivity to local noise restricts their predictive reliability when applied to unseen data. Furthermore, the clear difference in accuracy between the two stations, with Toilchun consistently yielding higher R and NSE values than Hwangji, suggests a lack of model transferability.

### Diagrammatic analysis based on visualization of the results

To gain a clearer insight into model performance, several visualization techniques were applied to complement the statistical evaluation. Scatter, violin, and box plots were first used to examine the distribution and agreement between measured and predicted COD values. These visual tools supported the quantitative results presented in Tables 4 and 5. Furthermore, Taylor diagrams were employed to summarize the correlation and standard deviation of each model relative to the observed data. In contrast, Circos and Chord diagrams helped illustrate the complex interrelationships among variables. All visual analyses correspond to the validation datasets to ensure consistent comparison.

**Fig. 4:**
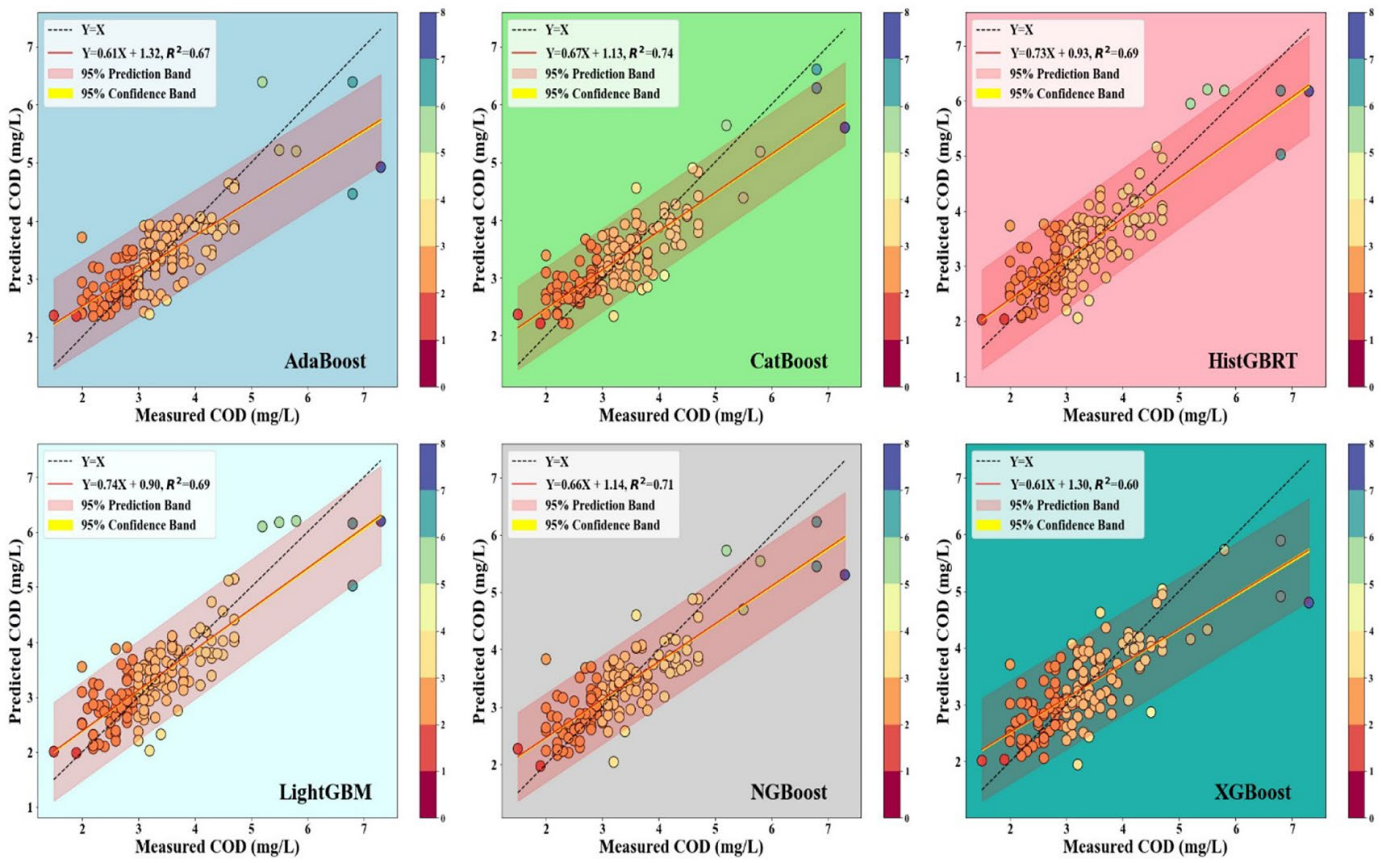
Scatterplots of the six applied boosting models for the Hwangji station.

**Fig. 5:**
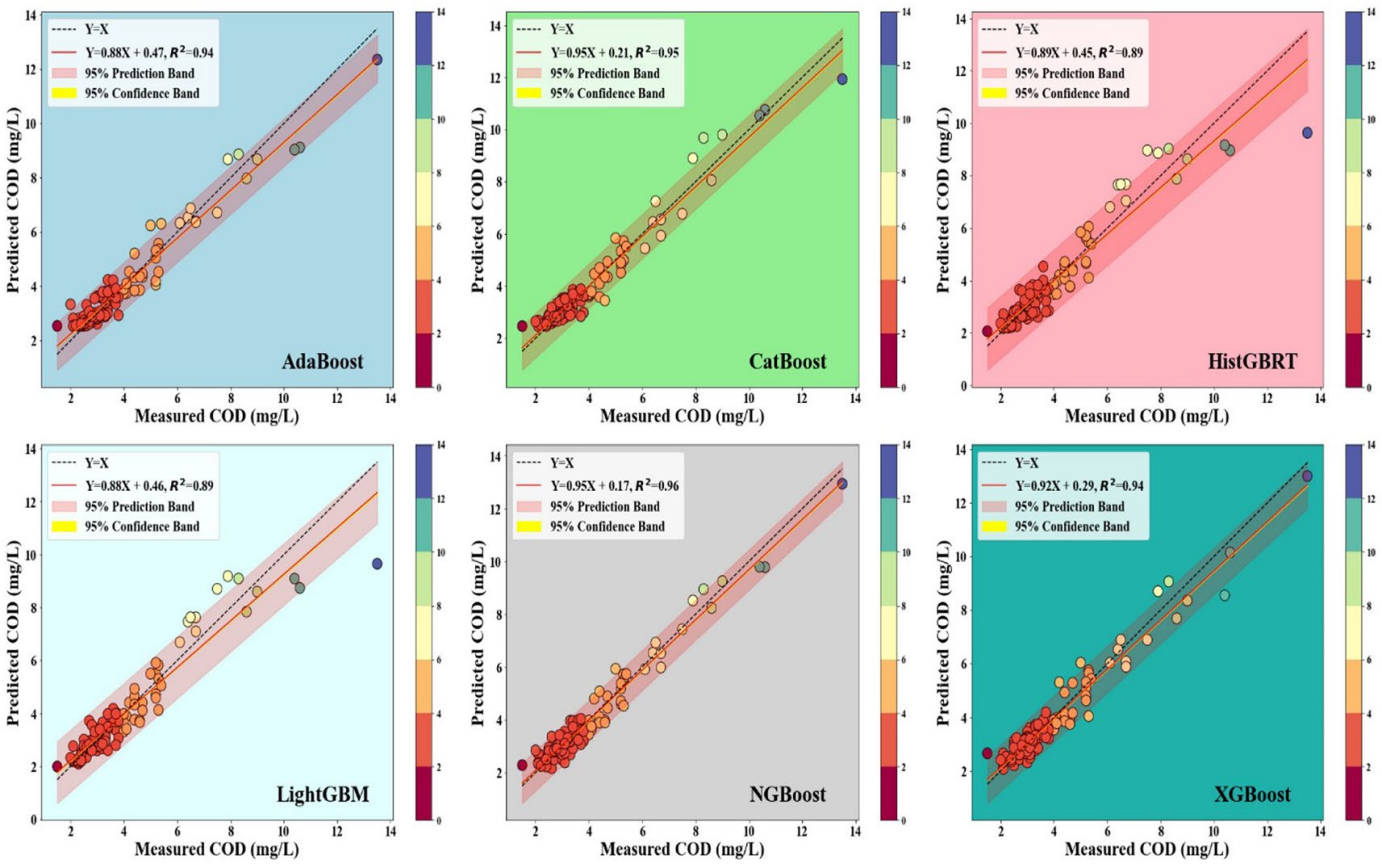
Scatterplots of the six applied boosting models for the Toilchun station.

Figures 4 and 5 display the scatter plots for the Hwangji and Toilchun stations, showing that CatBoost achieved the strongest agreement with the observed COD values, yielding the highest coefficients of determination (R^2^ ≈ 0.71 for Hwangji and ≈ 0.95 for Toilchun). CatBoost and XGBoost models also demonstrate higher accuracy in Toilchun (R^2^ ≈ 0.948 and ≈0.942, respectively) compared to the Hwangji Station (R^2^ ≈ 0.742 and ≈0.599, respectively). However, LightGBM, AdaBoost, and HistGBRT show moderate accuracy, with lower R^2^ values than NGBoost and CatBoost, especially at Hwangji Station. As for the Toilchun Station, the dispersion of the predicted values (dots in the scatter plots) aligns closely with the 1:1 line, indicating more accurate modeling. Also, comparing the slope values of the trendlines for the Hwangji (0.605 < m < 740) and Toilchun (0.891 < m < 0.951) stations shows that the predicted COD values are closer to the measured values. Consequently, it can be clearly concluded that the predictions are more accurate for Toilchun Station, potentially due to lower correlations among the independent variables and COD at Hwangji Station (refer to the correlation values provided in Tables 1 and 2).

In the next step, the accuracy of the six applied boosting methods in predicting COD was analyzed using boxplots for the two stations. As indicated by the lower whiskers of the boxes, none of the models captured the minimum COD values. It can be observed that the XGBoost model performed slightly better than the others in this regard. The systematic underprediction of minimum COD values for all boosting algorithms suggests an intrinsic model bias toward central tendency conditions (see the PBIAS results contained in Tables 4 and 5). This limitation, clearly visible in the lower whiskers of the boxplots and the lower tails of the violin plots, indicates that the models are more effective at capturing average pollution levels than predicting very low COD concentrations. Specifically, the positive PBIAS values observed for several models, such as NGBoost5 (PBIAS = 0.521%) at Hwangji and XGBoost7 (PBIAS = 1.353%) at Toilchun, further quantify this tendency to deviate from the observed extremes. This matter confirms that while the models are globally accurate, they maintain a slight systematic offset when dealing with the full range of hydrological variability.

**Fig. 6:**
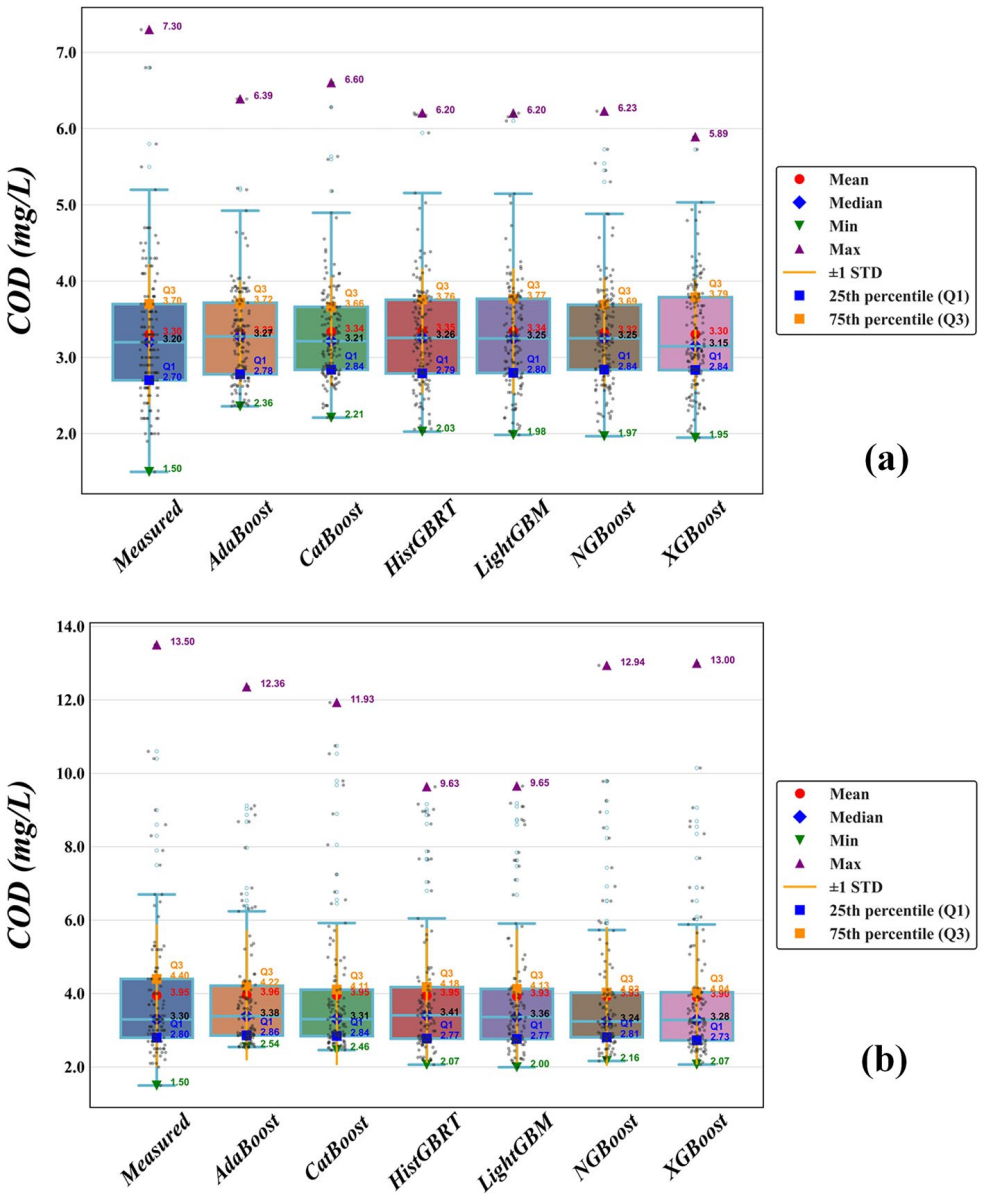
Boxplots of the measured and predicted COD values from different boosting models in the validation set, (**a**) Hwangji station; (**b**) Toilchun station.

For Hwangji Station (Fig. 6a), which has a lower COD range (approximately 2–6 mg/L), CatBoost and LightGBM align well with the median (the middle line of each box) and exhibit fewer outliers. At Toilchun station (Fig. 6b), the models closely match the median COD value, but the body of the boxes shows varying spreads. Overall, CatBoost and NGBoost provided a smaller body box in comparison to the measured values; however, they acted fairly in predicting extreme values.

**Fig. 7:**
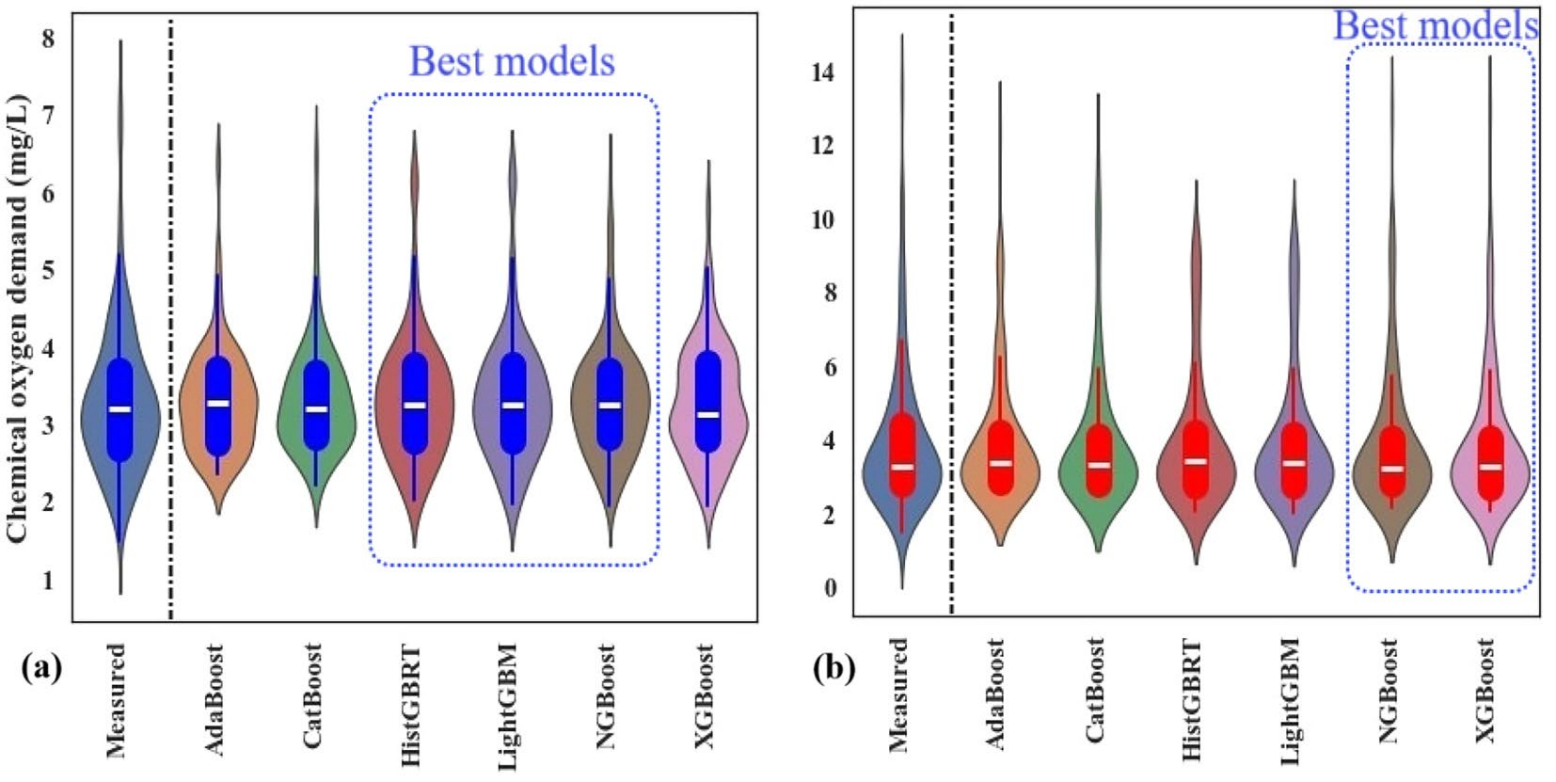
Depicted violin graphs of the measured and predicted COD values from different boosting models in the validation set, (**a**) Hwangji station; (**b**) Toilchun station.

Figure 7 illustrates the violin plots for COD predictions at the Hwangji and Toilchun stations. These plots depict the distribution of COD data using density curves. As in the boxplots (Fig. 6), each density curve has a small box plot at its center showing the ends of the 1st and 3rd quartiles and the median. However, unlike boxplots, violin plots do not distinguish between outliers and extreme values. Considering the median lines of the applied models at both stations, the predicted data aligned well with the measured COD data. At Hwangji Station (Fig. 7a), where COD levels range from 1 to 8 mg/L, AdaBoost and CatBoost struggled to predict the lower COD values (COD < 2 mg/L). In general, HistGBRT, NGBoost, and LightGBM models show more similar density curves to the measured values. At the Toilchun Station (Fig. 7b), COD concentrations reached up to 14 mg/L. NGBoost and XGBoost models exhibited broader distributions, indicating a stronger ability to represent high-end or extreme COD values. This trend was further supported by the presence of additional outliers in the corresponding boxplots (Fig. 6b).

**Fig. 8:**
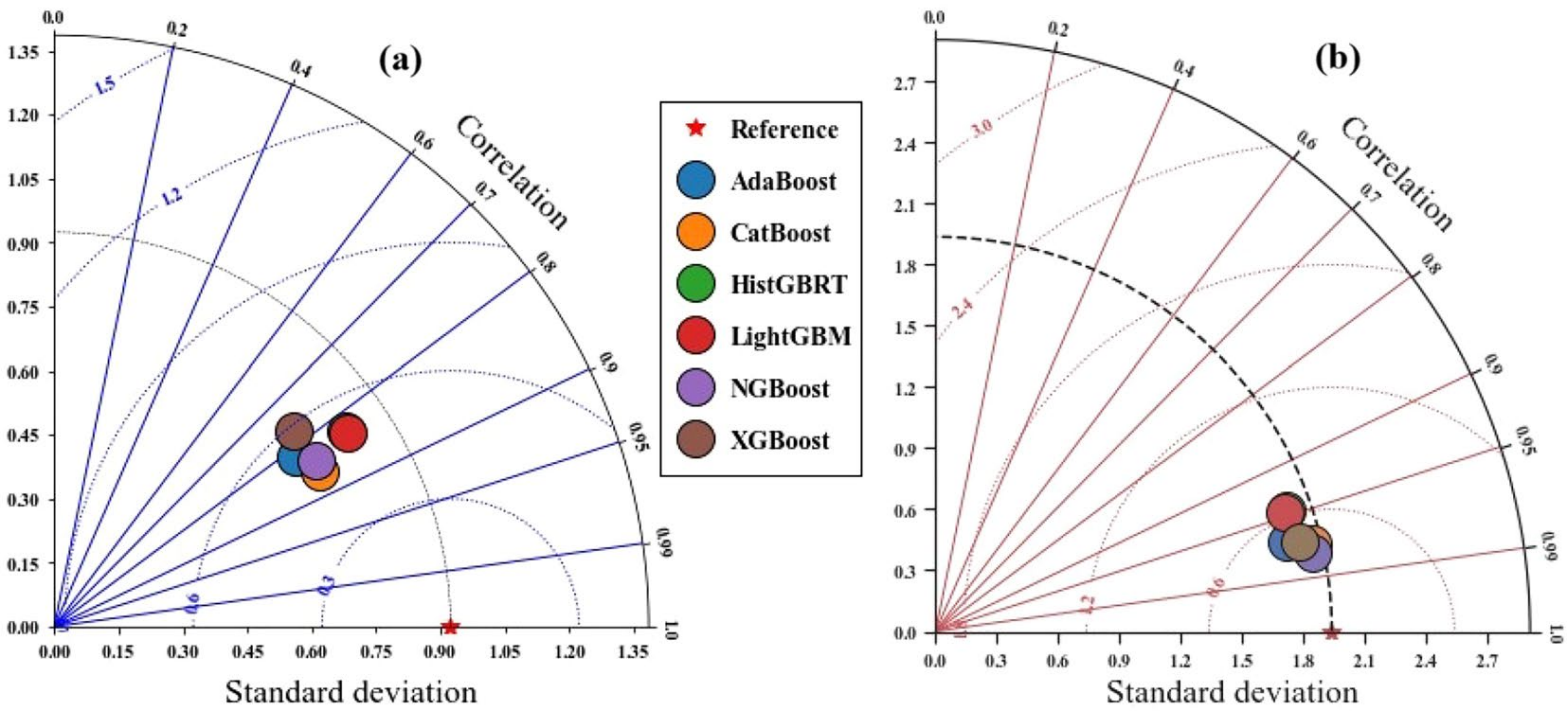
Taylor diagrams showing the performance of the boosting models: (**a**) Hwangji station; (**b**) Toilchun station.

Figure 8 presents Taylor diagrams summarizing the accuracy of the machine learning models for COD prediction at both stations. At Hwangji (Fig. 8a), most models achieved strong correlations with observed values (R > 0.75). Among them, CatBoost and LightGBM were positioned closest to the reference point, indicating superior predictive accuracy and stability. AdaBoost, on the other hand, displayed a lower standard deviation, reflecting a narrower prediction range. For Toilchun Station, the models formed a tighter cluster, indicating consistent behavior across algorithms. Nevertheless, NGBoost stood out as the best-performing model, with predicted values aligning more closely with the measured COD than at Hwangji Station.

**Fig. 9:**
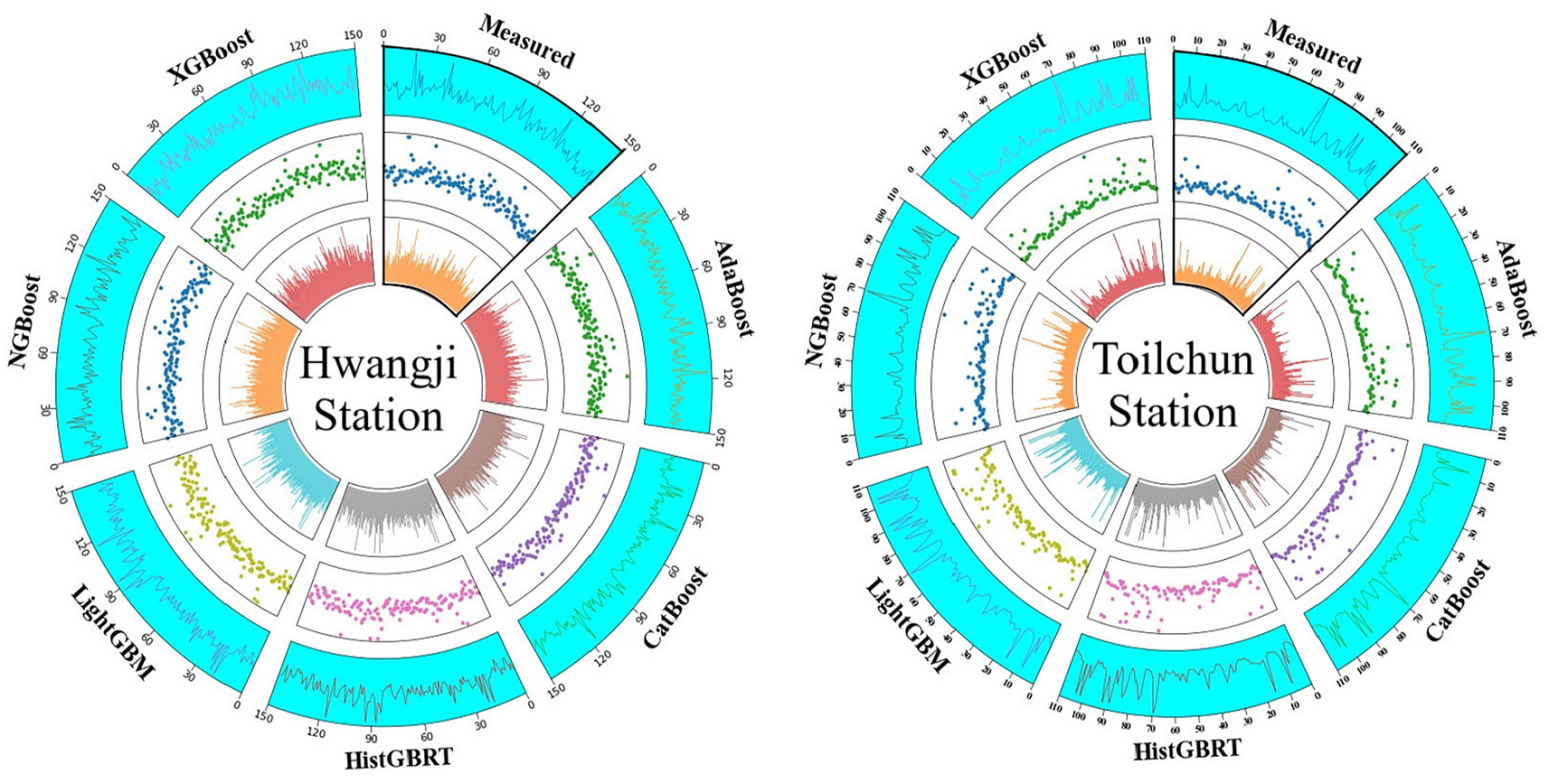
Circos Plot for circular visualization of the models’ performances.

Figure 9 shows the Circos plots, which provide an overall visualization of the boosting models’ performance at the Hwangji and Toilchun stations. The radial layout of these plots allows a direct comparison between observed and predicted COD values, illustrating each model’s accuracy and variability across the validation data. Each diagram consists of three concentric rings representing the density distribution, the spread of individual predictions, and the temporal pattern of the COD series. Among the tested models, XGBoost and CatBoost displayed the closest agreement with the observed data, indicating their strong ability to reproduce COD dynamics. Differences in the density and spread of points within the inner rings further reflect how sensitively each model responds to variations in COD levels. For example, in both stations, HistGBRT and LightGBM display more noticeable deviations compared to models like AdaBoost and CatBoost models.

**Fig. 10:**
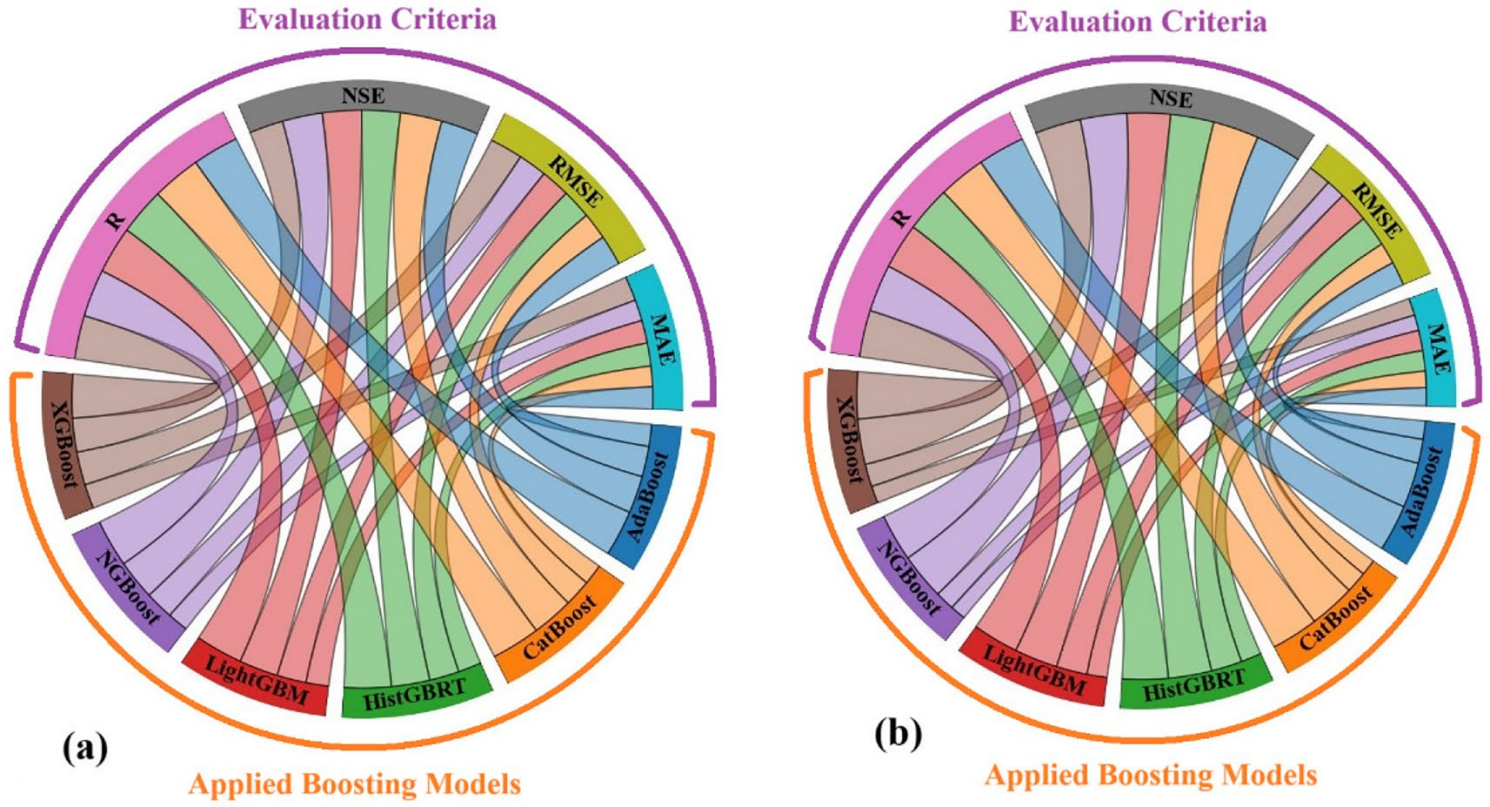
Interrelationships for models’ comparison using Chord Diagram, (**a**) Hwangji station; (**b**) Toilchun station.

The chord diagrams, presented in Fig. 10, illustrate the relationships between the applied boosting models, and statistical measures including R, NSE, RMSE, and MAE, for the predicted COD values at both the Hwangji and Toilchun stations. In these diagrams, each chord band represents a contribution of a specific model to a performance metric, assisting with a visual judgment of the effectiveness and accuracy of each model. In the chord diagram for Hwangji Station (Fig. 10a), the NGBoost model shows a strong association with NSE. CatBoost is also well-represented by high NSE and R values, indicating good generalization to the validation data. XGBoost shows a clear relationship with the correlation coefficient but also exhibits a drawback: a tendency to produce higher RMSE values, which can be a sign of overfitting. AdaBoost model displays a balanced relationship between NSE and RMSE. This matter suggests a steady prediction, but less optimal performance compared to NGBoost and CatBoost models. For the Toilchun Station (Fig. 10b), compared to the other models, XGBoost and CatBoost demonstrate slightly stronger ties to high R and NSE values, indicating its ability to achieve a high degree of correlation and similarity between the predicted and observed COD values. Based on the narrower chords of NGBoost to the RMSE and MAE values, it can be concluded that this model outperformed the other applied models, highlighting its consistency across performance metrics. CatBoost and LightGBM show moderate associations with NSE. Overall, the chord diagrams in Fig. 10 emphasize that NGBoost and CatBoost offer a good balance between predictive accuracy and similarity.

### Results analysis using Shapley additive explanations (SHAP)

**Fig. 11:**
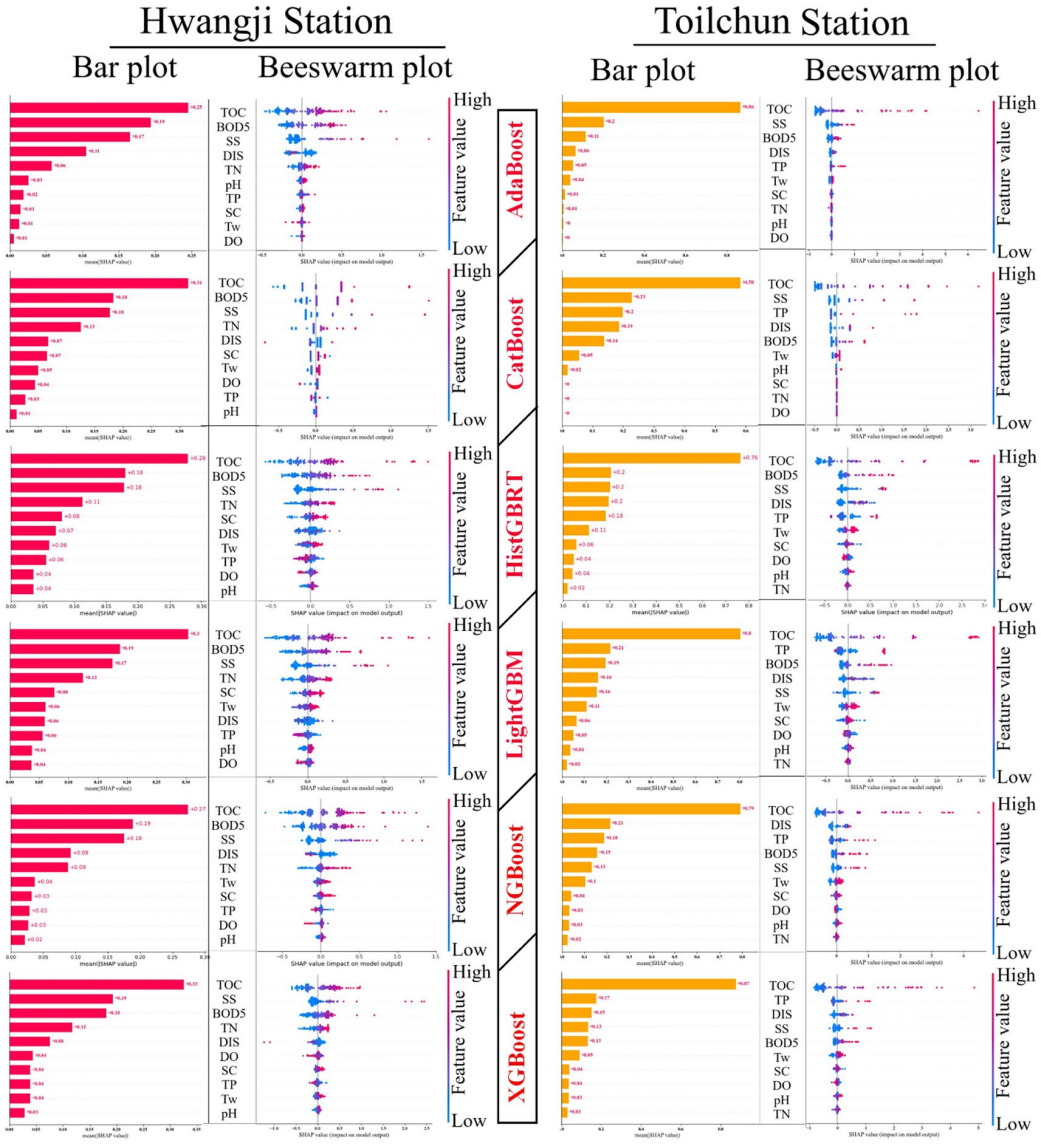
Features ranking and Global models explainability using SHAP for the Hwangji and Toilchun stations in terms of Bar plots (Mean SHAP value of features) and Beeswarm plots (SHAP global explanation), The input variables (i.e., features) were ranked according to their mean absolute SHAP values, as shown on the x-axis.

The ability to understand an ML model is crucial, as it allows users to interpret its processes and make informed decisions^57^ introduced SHAP, an interpretability framework grounded in Shapley’s cooperative game theory. In this study, SHAP was applied to interpret the most accurate predictive model and to better understand the contribution of each input variable to the model output. The SHAP method provides a transparent way to explain model decisions without compromising predictive accuracy. As noted by^58^, SHAP uniquely satisfies three desirable properties for interpretability—consistency, missingness, and local accuracy. It calculates Shapley values as indicators of feature importance, quantifying how each input influences an individual prediction. In essence, the method compares the model output when a feature is included versus when it takes a baseline (mean) value, thereby estimating the marginal contribution of that feature to the overall prediction^59^. Each input feature’s contribution to the model output is assessed by SHAP, which also determines whether the contribution is positive or negative. In the meantime, SHAP can determine each feature’s contribution to each anticipated output^60^. Figure 11 provides a comprehensive view of the feature importance for COD prediction at two stations using SHAP-based feature ranking.

The mean absolute SHAP value for each variable is shown in the bar plot in Fig. 11. The importance of each feature is determined by the mean of its absolute Shapley values, with features listed along the y-axis. The distribution of SHAP values for the dataset is presented in Fig. 11 as a beeswarm plot, where each point represents a predicted outcome. SHAP values are displayed on the x-axis, and color coding represents feature values, with red indicating higher values and blue indicating lower values. Plot colors reflect changing values, as indicated by the color scale bars on the right side of the figures. In the SHAP plots, positive values indicate that a feature increases the model’s predicted COD, while negative values represent a decreasing effect. Among all input variables, TOC, BOD₅, and SS consistently show positive SHAP contributions, demonstrating their strong influence on the model output. Across all models, TOC is the most influential predictor, indicating that higher organic carbon concentrations are strongly associated with higher COD levels. This consistent relationship underscores the critical role of TOC in driving COD variability within the studied river systems.

From a physico-chemical perspective, the dominance of TOC, BOD_5_, and SS is highly significant. The measures of TOC and BOD_5_ both represent organic carbon content and its various states. In addition to this relationship, both TOC and BOD_5_ are critical elements of each component of COD’s total oxygen demand. SS, the most significant volume of COD, represents the sediment and debris brought into a river system as a result of extreme surface runoff created by high flows. Despite architectural differences, several boosting algorithms returned the same three variables (TOC, BOD5, and SS) as top predictors, indicating that the underlying process captured by these algorithms is consistent across a variety of boosting algorithms and does not simply reflect statistical correlations.

At the Hwangji Station, followed by TOC, BOD_5_, and SS had the most influence on the prediction of COD values. Therefore, BOD_5_ and SS are also among the high-ranking features that substantially influence COD levels in this area. At the Toilchun Station, in addition to TOC, other independent parameters, such as SS, BOD5, TP, and DIS, emerged as the most effective variables for predicting COD. Among the applied boosting models, the AdaBoost and CatBoost models show a similar ranking pattern at both stations, with TOC, BOD_5_, and SS standing out at Hwangji, while TOC, TP, and BOD_5_ are more critical for Toilchun. While TOC and BOD_5_ reflect the organic load of the water and its impact on COD, SS is an indicator for a COD contribution from suspended sediments and organics attached to it, that originate from surface flow and erosion. The parameter TP (total phosphorus) represents the contribution of surface erosion or treated wastewater without sufficient phosphorus removal. On the other hand, HistGBRT, LightGBM, NGBoost, and XGBoost highlight TOC as the primary independent variable but show slight variations in the importance of other input parameters. In general, in the presence of variables such as TOC, BOD5, SS, and TN, other input variables, such as pH, TP, SC, Tw, and DO, do not directly impact the prediction of COD. This claim can also be supported by the fact that input parameters like pH, DO, TO, Tw, and DIS were neglected in HistGBRT5, LightGBM5, XGBoost, and NGBoost5 were chosen as the optimal boosting models for the Hwangji Station (see Tables 3 and 4). In addition, at the Toilchun Station, some best predicted boosting models were constructed based on the 7th input vectors (see Table 3) such as AdaBoost7, NGBoost7, and XGBoost7 (see Table 4), which indicates that only SS, TN, TOC, and SC were the effective independent parameters.

The findings of SHAP show that there are large differences between the two locations regarding the relative importance of features within the model. Organic indicators are still the main drivers of both sites, but the relative importance of discharge and total phosphorus is higher at Toilchun than at Hwangji. This difference indicates that the Toilchun station may be more affected by non-point source pollution, such as agricultural runoff, and that discharge acts as a means of transporting nutrients and organic-rich sediments. In contrast, the more localized influence of SC and SS at the Hwangji site indicates that the pollution source may be more related to industrial or other stable point-source waste. The differences in importance between features at the two sites also highlight the necessity for localized interpretation in developing appropriate water quality management strategies.

Alongside the bar plot in Fig. 11, the beeswarm plots provide a detailed summary of how the top features in the dataset influence the model’s output. In these plots, each dot represents a sample and is colored by feature value (red for higher values, blue for lower values), allowing the relationship between feature values and their impact on COD predictions to be observed. As expected, the beeswarm plots for TOC show that higher values consistently lead to greater SHAP impacts across both stations, indicating a positive correlation between TOC and COD. This is further confirmed by the correlation values in Table 1, where TOC exhibits the highest correlation with COD (R = 0.944).

**Fig. 12:**
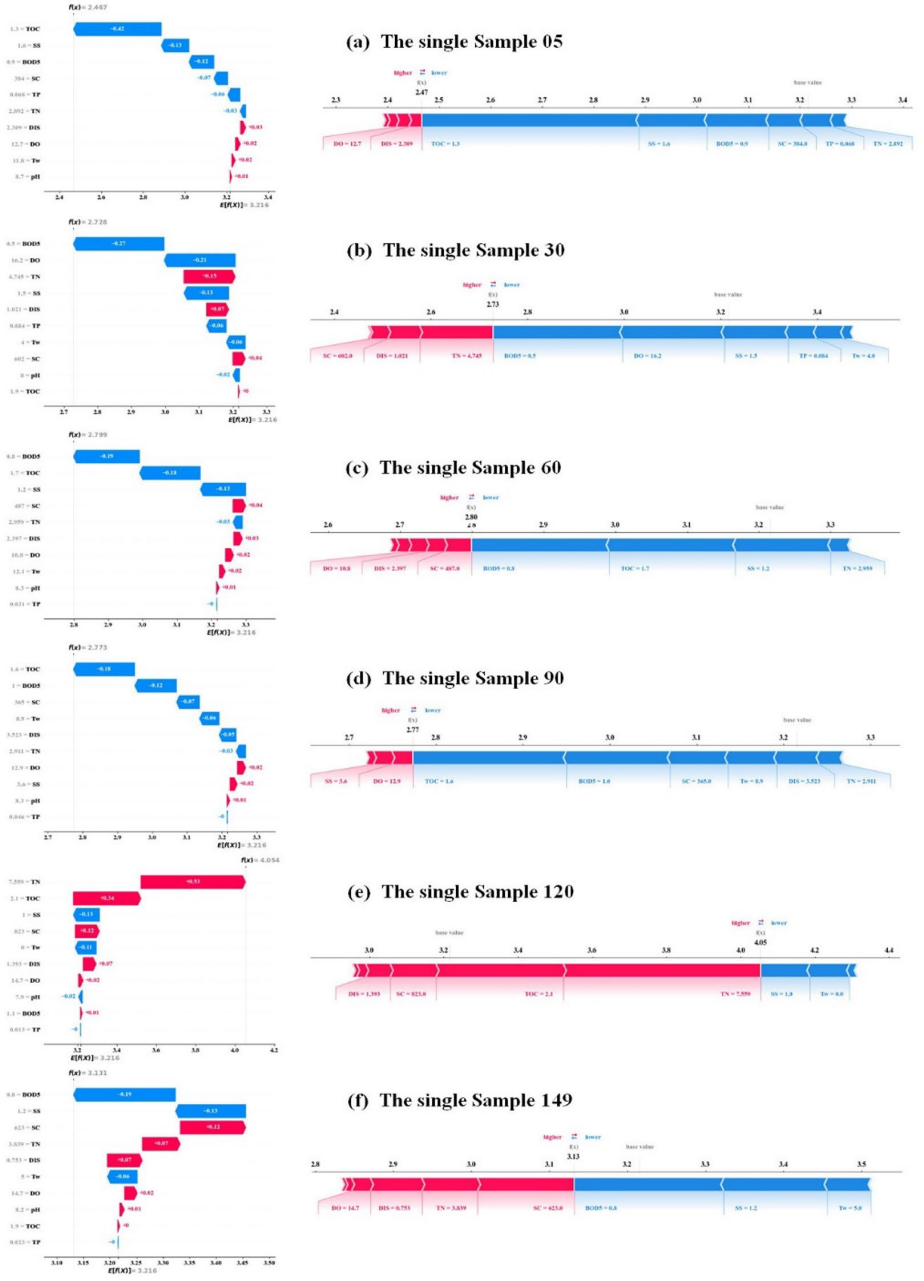
SHAP local explanation for the Hwangji station, Left panel: SHAP waterfall plot on selected, Right panel: SHAP force plot on selected samples.

**Fig. 13:**
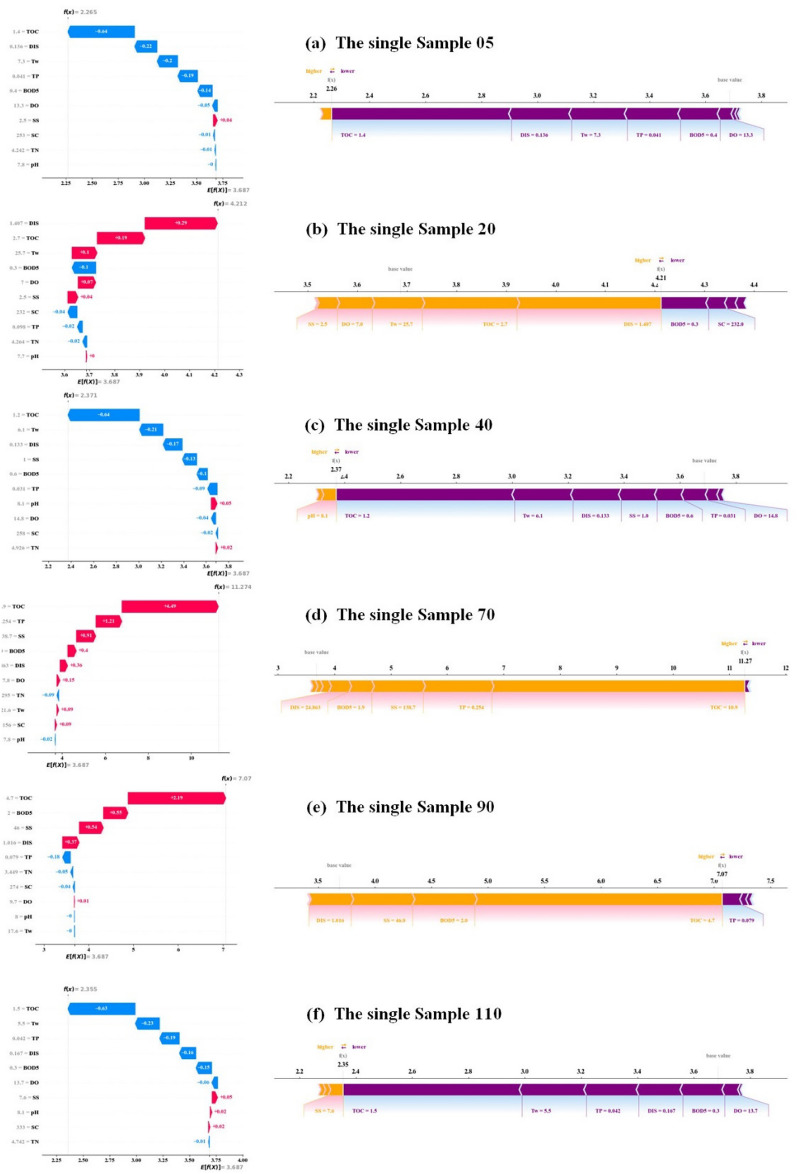
SHAP local explanation for the Toilchun station, Left panel: SHAP waterfall plot on selected, Right panel: SHAP force plot on selected samples.

Figures 12 and 13 illustrate the local interpretability of COD predictions for the two stations (Hwangji and Toilchun) using SHAP. Each Figure includes waterfall plots (left panels) and force plots (right panels) for individual samples, depicting the contributions of different parameters to the model output. All samples’ SHAP waterfall plots are displayed in Fig. 12 for the Hwangji Station, which interprets each variable’s distinct contribution to the outcome at any given point. In this figure, the baseline, or average expected value, is E[f(x)], while the final prediction is f(x). Each row’s SHAP values indicate how individual features interact and contribute to the final predicted value. Positive feature contributions are shown in red, while negative contributions are indicated in blue. While features with a negative influence helped reduce the output, features with a positive impact helped provide strong predictive results. Overall, the two factors that most significantly affect prediction outcomes are TOC and DIS. However, this order of significance varies for various samples: (a) TOC has gr negative impact, (b) DIS has the highest positive impact, (c) TOC has the highest negative impact, (d) TOC has the most significant positive influence, (e) TOC shows a positive impact, (f) TOC has the most significant negative influence. Figure 12 presents the SHAP force plot, which can be interpreted as a horizontal projection of the waterfall plot, highlighting features that drive predictions higher (orange) or lower (purple).

Figure 13 presents the SHAP waterfall plot for Toilchun Station, which illustrates each variable’s distinct contribution to the predicted outcome. The baseline, or average expected value, is $E[f(x)]$, while the final prediction is $f(x)$. Each row’s SHAP values show how individual features interact and contribute to the final predicted value. Positive contributions are shown in red, and negative contributions are shown in blue. Features with negative influences reduce the output, whereas features with positive influences enhance prediction. Overall, TOC, BOD5, and TN exerted the most significant influence on prediction outcomes, though the order of importance varies across samples: (a) TOC has the highest negative impact, (b) BOD_5_ has the highest negative influence, (c) BOD_5_ has the highest negative impact, (d) TOC has the highest negative impact, (e) TN has the highest positive impact, (f) BOD_5_ has the highest negative impact. The SHAP force plot in Fig. 13 serves as a horizontal projection of the waterfall plot, highlighting features that increase the prediction (red) or decrease it (blue).

The application of ensemble learning methods demonstrates high potential in modeling complicated hydro-environmental parameters such as COD. Since ensemble models integrate the predictions of multiple weak learners, they tend to achieve better accuracy and robustness than single-model approaches. According to the obtained results, it can be reported that the gradient boosting models (e.g., XGBoost, LightGBM, HistGBRT) achieved higher similarity measures (i.e., R and NSE) values and lower deviance error metrics (i.e., RMSE and MAE) in training, but adaptive models like NGBoost and CatBoost offered better stability in the validation set (see Tables 4 and 5). This outcome suggests that, while gradient boosting models may fit the training data closely, adaptive methods performed better in handling variability within the dataset. Additionally, models using more comprehensive qualitative input variables such as SS, TN, TOC, SC, and BOD_5_ (Table 3) tend to perform better, with lower RMSE and higher R values compared to those using fewer variables. This indicates that the diversity of inputs plays a crucial role in capturing the complex relationships necessary for accurate COD prediction. However, it should be noted that including all available input variables (as shown in the 8th and 9th model configurations in Table 3, e.g., AdaBoost8 and AdaBoost9) did not yield the most accurate COD predictions.

Overall, the outcomes of this research demonstrated the high ability of boosting ML models for predicting water quality parameters such as COD. However, there are some limitations in this study worth mentioning. Firstly, the dataset employed in this study may not fully capture the seasonal or long-term variations in water quality parameters. This limitation may have influenced the model’s capability to generalize across different temporal conditions. Future research could enhance model robustness by incorporating longer and seasonally diverse datasets that capture the temporal variability of COD dynamics. Another constraint arises from the limited range of water quality parameters available for analysis. Although this was determined by the existing monitoring program, it remains an important consideration. While TOC, BOD_5_, and SS were identified as the most influential predictors, incorporating additional parameters such as other nitrogen and phosphorus species or microbial indicators could further enhance model accuracy and provide a more comprehensive understanding of COD dynamics. Moreover, future studies might explore alternative ensemble strategies, including bagging^61^, Dagging^62^, and stacking^63^, to potentially enhance predictive performance beyond that achieved with boosting algorithms.

In terms of practicality, the confirmation that TOC is the most important predictor of COD suggests that water quality degradation in South Korean rivers is largely caused by organic loading. Therefore, using TOC as a means of monitoring COD may provide valuable information regarding water quality changes over time. Overall, Recent developments in the field of water quality modeling indicate that the current popularity of ensemble boosting appears to give better predictive accuracy than traditional methods, including neural networks, when dealing with the tabular format of data that water quality models typically require.

## Conclusion and future research

This study evaluated the performance of six boosting-based ensemble machine learning models: AdaBoost, CatBoost, XGBoost, HistGBRT, LightGBM, and NGBoost. These models were used to predict COD at two long-term monitoring stations in South Korea. Based on various statistical performance measures, including R, NSE, RMSE, MAE, and PBIAS, NGBoost and CatBoost showed better predictive ability than the other models, especially in validation datasets. Although XGBoost performed almost perfectly during training, its lower validation accuracy raised concerns about overfitting. This highlights the need for thorough model evaluation that goes beyond training accuracy.

In addition to predictive accuracy, this study focused on model interpretability using SHapley Additive exPlanations. The SHAP analysis consistently identified TOC as the main factor influencing COD variability, followed by BOD_5_, SS, and DIS. These findings are meaningful and align well with established biochemical and hydrological processes that control organic pollution in rivers. The consistency between SHAP insights and known water quality processes boosts confidence in the explanatory reliability of the proposed models.

However, there are some limitations to consider. First, while NGBoost provides probabilistic predictions, it assessed uncertainty implicitly through predictive distributions. This approach did not include explicit uncertainty propagation or confidence interval validation against independent observations. Although NGBoost’s framework is useful for risk-informed decision-making, future studies should explicitly evaluate prediction intervals, coverage probability, and uncertainty calibration to better support water management decisions.

Second, the models developed are specific to particular stations and were trained and validated using historical data from two monitoring sites. This means we cannot directly transfer them to other basins or climatic regions without recalibration or external validation. The strong performance observed at Toilchun and Hwangji stations reflects local hydro-environmental features, making spatial generalization a challenge for future research. Incorporating multi-site or cross-basin validation strategies would be essential to assess model reliability on a larger scale.

Third, although the proposed framework shows promise for operational use, this study did not explicitly test real-time applicability. Practical use would require continuous access to key predictors like TOC and BOD_5_, which might need high-frequency sensors or reliable surrogate measurements. Sensor noise, missing data, and latency issues could also impact real-time performance. Thus, while the results suggest potential for monitoring and early warning systems, future work should examine model performance under real-time data limitations and streaming conditions.

In summary, NGBoost and CatBoost strike a good balance between predictive accuracy, robustness, and interpretability for estimating COD. By combining explainable boosting models with SHAP, the study offers accurate predictions and clear insights into the main drivers of water quality changes. Future research should focus on uncertainty quantification, cross-site generalization, and real-time application to enhance the practical value of explainable machine learning frameworks for water quality monitoring and environmental decision support.

## Supplementary Information

Supplementary Information.

## Data Availability

The data presented in this study will be available on interested request from the corresponding author.

## Abbreviations

AdaBoost: Adaptive boosting
AI: Artificial intelligence
ANN: Artificial neural network
Bi-LSTM: Bidirectional long short-term memory
BOD_5_: Biochemical oxygen demand
CARTs: Classification and regression trees
CatBoost: Categorical boosting
COD: Chemical oxygen demand
Cv: Variation coefficient
DIS: Station discharge
DL: Deep learning
DO: Dissolved oxygen
EFB: Exclusive feature bundling
GBDT: Gradient boosting decision tree
GOSS: Gradient-based one-side sampling
HFCWs: Horizontal flow constructed wetlands
HistGBRT: Histogram gradient boosting
LightGBM: Light gradient boosting
LSTM: Long short-term memory
MAE: Mean absolute error
ML: Machine learning
MLP: Multilayer perceptron
MLR: Multiple linear regression
NGBoost: Natural gradient boosting
NSE: Nash–Sutcliffe efficiency
PBIAS: Percent bias
pH: Potential of hydrogen
R: Correlation coefficient
R^2^: Coefficients of determination
RF: Random forest
RMSE: Root-mean-square error
RNN: Recurrent neural network
SC: Electrical conductivity
SHAP: SHapley Additive exPlanations
SS: Suspended solids
SVR: Support vector regression
SD: Standard deviation
TN: Total nitrogen
TOC: Total organic carbon
TP: Total phosphorus
Tw: Water temperature
XGBoost: Extreme gradient boosting
X_max_: Maximum value
X_mean_: Average value
X_min_: Minimum value
