# Supplementary material for: Accurate and interpretable prediction of chemical oxygen demand using explainable boosting algorithms with SHAP analysis
Source: Sci Rep. 2026 Feb 13;16:6359. doi: 10.1038/s41598-026-38757-4 (PMC12905304; doi:10.1038/s41598-026-38757-4)
Supplement: Supplementary file 1 — Supplementary Information. [file 41598_2026_38757_MOESM1_ESM.docx]

**Accurate and Interpretable Prediction of Chemical Oxygen Demand Using Explainable Boosting Algorithms with SHAP Analysis**

***Supplementary Information***

***Contents of Supplementary Information***

Table S1. 10-Fold Cross-Validation Results for COD Modelling: CatBoost Model.

Table S2. 10-Fold Cross-Validation Results for COD Modelling: XGBoost Model.

Table S3. 10-fold cross validation results for predicting COD utilizing the best AdaBoost model.

Table S4. U95 Uncertainty Analysis Summary.

Table S5. Optimal hyperparameters and tuning ranges for the evaluated machine learning models.

Table S6. Kruskal–Wallis Test Results for Model Performance at Hwangji and Toilchun Stations.

**Figure S1. 10-fold cross-validation results for COD prediction using the CatBoost model: Hwangji station.**

**Figure S2. 10-fold cross-validation results for COD prediction using the XGBoost model: Toilchun station.**

Figure S3. Cosine Amplitude Sensitivity Analysis: HWANGJI Station.

Figure S4. Cosine Amplitude Sensitivity Analysis: TOILCHUN Station.

Figure S5. U₉₅ uncertainty analysis and models ranking: bar plot left panel and spider plot right panel: HWANGJI Station.

Figure S6. U₉₅ uncertainty analysis and models ranking: bar plot left panel and spider plot right panel: TOILCHUN Station.

Figure S7. **Training and Validation Learning Curves with Generalization Diagnostics.**

**Figure S8.** Statistical comparison of model performances at Hwangji station based on p-values from the Kruskal–Wallis test.

**Figure S9.** Statistical comparison of model performances at Toilchun station based on p-values from the Kruskal–Wallis test.

Table S1. 10-Fold Cross-Validation Results for COD Modelling: CatBoost Model

| Folds |  | Results for **Hwangji Station** | | | |  |
| --- | --- | --- | --- | --- | --- | --- |
|  |  | RMSE  (mg/L) | MAE  (mg/L) | R | NSE |  |
| Fold 1 |  | 0.549 | 0.378 | 0.957 | 0.852 |  |
| Fold 2 |  | 0.425 | 0.351 | 0.810 | 0.653 |  |
| Fold 3 |  | 0.404 | 0.325 | 0.837 | 0.691 |  |
| Fold 4 |  | 0.525 | 0.376 | 0.937 | 0.843 |  |
| Fold 5 |  | 0.445 | 0.351 | 0.782 | 0.604 |  |
| Fold 6 |  | 0.458 | 0.365 | 0.635 | 0.379 |  |
| Fold 7 |  | 0.472 | 0.365 | 0.884 | 0.733 |  |
| Fold 8 |  | 0.381 | 0.295 | 0.841 | 0.690 |  |
| Fold 9 |  | 0.562 | 0.442 | 0.842 | 0.709 |  |
| Fold 10 |  | 0.612 | 0.363 | 0.808 | 0.600 |  |
| **Mean** |  | **0.483** | **0.361** | **0.833** | **0.675** |  |
| **SD** |  | **0.076** | **0.038** | **0.089** | **0.135** |  |

Table S2. 10-Fold Cross-Validation Results for COD Modelling: XGBoost Model

| Folds |  | Results for **Toilchun Station** | | | |  |
| --- | --- | --- | --- | --- | --- | --- |
|  |  | RMSE  (mg/L) | MAE  (mg/L) | R | NSE |  |
| Fold 1 |  | 0.387 | 0.332 | 0.907 | 0.807 |  |
| Fold 2 |  | 0.710 | 0.413 | 0.910 | 0.785 |  |
| Fold 3 |  | 0.628 | 0.402 | 0.908 | 0.813 |  |
| Fold 4 |  | 0.481 | 0.399 | 0.984 | 0.963 |  |
| Fold 5 |  | 0.346 | 0.277 | 0.944 | 0.839 |  |
| Fold 6 |  | 0.357 | 0.268 | 0.957 | 0.915 |  |
| Fold 7 |  | 0.494 | 0.369 | 0.937 | 0.876 |  |
| Fold 8 |  | 0.601 | 0.408 | 0.973 | 0.920 |  |
| Fold 9 |  | 0.338 | 0.265 | 0.986 | 0.971 |  |
| Fold 10 |  | 0.511 | 0.371 | 0.875 | 0.761 |  |
| **Mean** |  | **0.485** | **0.350** | **0.938** | **0.865** |  |
| **SD** |  | **0.130** | **0.060** | **0.038** | **0.075** |  |

Table S3. 10-fold cross validation results for predicting COD utilizing the best AdaBoost model

| Folds |  | Results for COD | | | |  |
| --- | --- | --- | --- | --- | --- | --- |
|  |  | RMSE  (mg/L) | MAE  (mg/L) | R | NSE |  |
| Fold 1 |  | 1.769 | 1.113 | 0.909 | 0.827 |  |
| Fold 2 |  | 1.345 | 1.065 | 0.910 | 0.827 |  |
| Fold 3 |  | 0.930 | 0.742 | 0.969 | 0.940 |  |
| Fold 4 |  | 1.181 | 0.906 | 0.957 | 0.915 |  |
| Fold 5 |  | 1.658 | 0.981 | 0.894 | 0.799 |  |
| Fold 6 |  | 1.123 | 0.894 | 0.953 | 0.909 |  |
| Fold 7 |  | 1.165 | 0.979 | 0.964 | 0.929 |  |
| Fold 8 |  | 1.061 | 0.912 | 0.952 | 0.905 |  |
| Fold 9 |  | 1.501 | 1.139 | 0.850 | 0.722 |  |
| Fold 10 |  | 1.455 | 0.946 | 0.904 | 0.818 |  |
| **Mean** |  | **1.319** | **0.968** | **0.926** | **0.859** |  |
| **SD** |  | **0.273** | **0.117** | **0.039** | **0.071** |  |

Table S4. U95 Uncertainty Analysis Summary.

| HWANGJI Station | | | | TOILCHUN Station | | |
| --- | --- | --- | --- | --- | --- | --- |
| N° | Models |  | U95_Test | N° | Models | U95_Test |
| 01 | CatBoost |  | 1.321 | 01 | NGBoost | 1.099 |
| 02 | NGBoost |  | 1.389 | 02 | CatBoost | 1.224 |
| 03 | LightGBM |  | 1.428 | 03 | XGBoost | 1.299 |
| 04 | HistGBRT |  | 1.446 | 04 | AdaBoost | 1.382 |
| 05 | AdaBoost |  | 1.492 | 05 | LightGBM | 1.756 |
| 06 | XGBoost |  | 1.620 | 06 | HistGBRT | 1.767 |

Table S5. Optimal hyperparameters and tuning ranges for the evaluated machine learning models.

| **Model** | **Hyperparameter** | **Tuning Range** | **Optimal Value** |
| --- | --- | --- | --- |
| AdaBoost | n_estimators | [50, 500] | 100 |
|  | learning_rate | [0.005, 0.3] | 0.2 |
|  | loss | linear, square, exponential | linear |
| CatBoost | iterations | [50, 500] | 100 |
|  | learning_rate | [0.005, 0.3] | 0.1 |
|  | depth | [1, 15] | 1 |
|  | l2_leaf_reg | [0.1, 10] | 0.2 |
| HistGBRT | max_iter | [50, 500] | 100 |
|  | max_depth | [1, 15] | 3 |
|  | learning_rate | [0.005, 0.3] | 0.1 |
| LightGBM | n_estimators | [50, 500] | 100 |
|  | learning_rate | [0.005, 0.3] | 0.05 |
|  | num_leaves | [20, 256] | 80 |
| NGBoost | n_estimators | [50, 500] | 150 |
|  | learning_rate | [0.005, 0.3] | 0.01 |
| XGBoost | n_estimators | [50, 500] | 100 |
|  | max_depth | [1, 15] | 3 |
|  | learning_rate | [0.005, 0.3] | 0.1 |
|  | gamma | [0, 1] | 0.5 |
|  | subsample | [0.5, 1] | 0.8 |

Table S6. Kruskal–Wallis Test Results for Model Performance at Hwangji and Toilchun Stations.

| **Models** |  | **Test statistic (H)** | | ***p*-value** | **𝐻_0_** | |
| --- | --- | --- | --- | --- | --- | --- |
| ***Hwangji* station** | | | | | | |
| **AdaBoost** |  | 0.6773 | | 0.4105 | *Fail to reject (Not significant)* | |
| **CatBoost** |  | 0.7418 | | 0.3891 | *Fail to reject (Not significant)* | |
| **HistGBRT** |  | 0.8275 | | 0.3630 | *Fail to reject (Not significant)*  *Fail to reject (Not significant)* | |
| **LightGBM** |  | 0.6962 | | 0.4041  0.3723 |  |  |
| **NGBoost** |  | 0.7960 | |  | *Fail to reject (Not significant)* | |
| **XGBoost** |  | 0.5089 | | 0.4756 | *Fail to reject (Not significant)* | |
| ***Toilchun* station** | | | | | | |
| **AdaBoost** |  | 0.1383 | 0.7100 | | | *Fail to reject (Not significant)* |
| **CatBoost** |  | 0.0046 | 0.9459 | | | *Fail to reject (Not significant)* |
| **HistGBRT** |  | 0.0026 | 0.9594 | | | *Fail to reject (Not significant)* |
| **LightGBM** |  | 0.0040 | 0.9493 | | | *Fail to reject (Not significant)* |
| **NGBoost** |  | 0.0380 | 0.8455 | | | *Fail to reject (Not significant)* |
| **XGBoost** |  | 0.0146 | 0.9039 | | | *Fail to reject (Not significant)* |

**
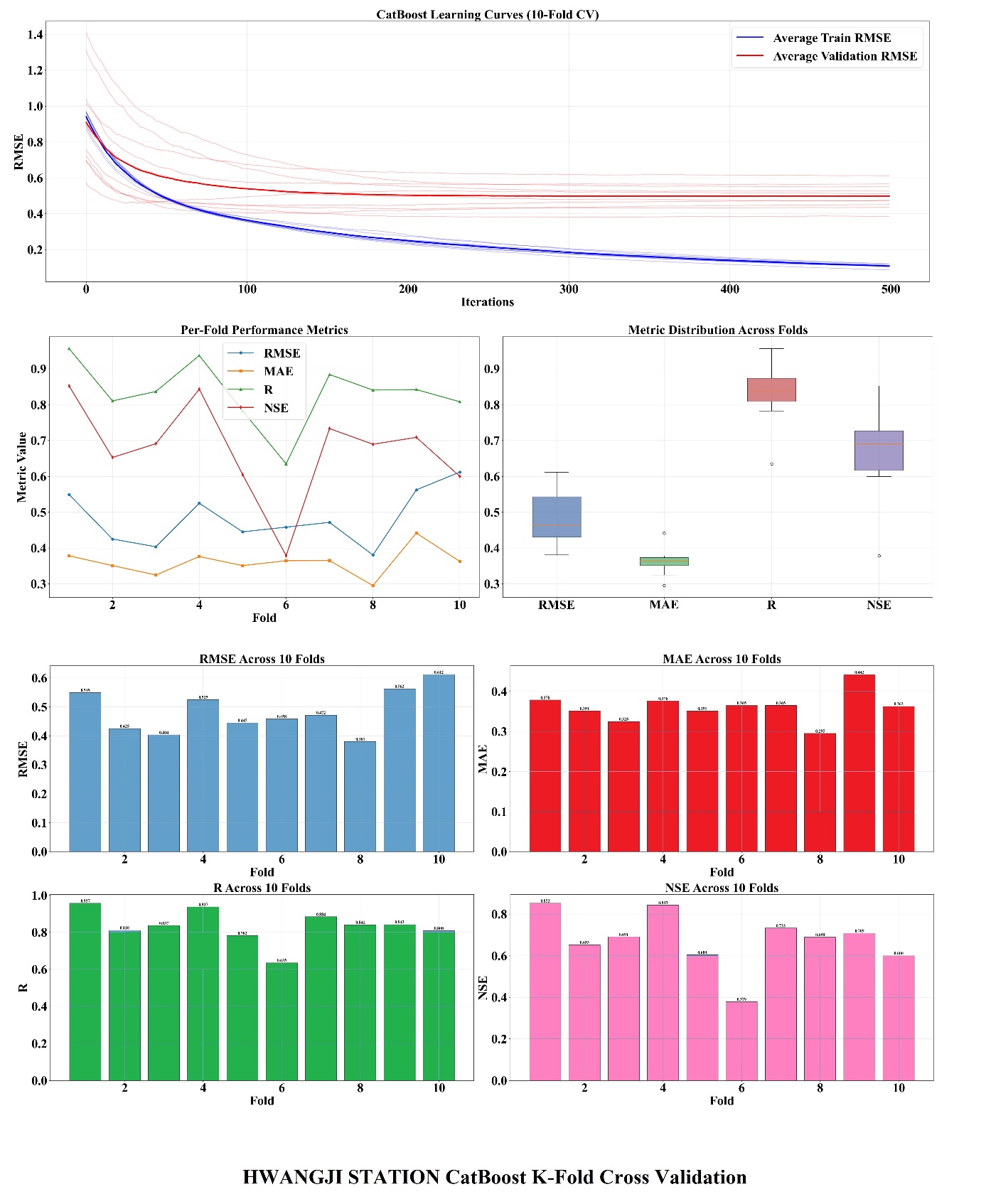
**

**Figure S1. 10-fold cross-validation results for COD prediction using the CatBoost model: Hwangji station.**

**
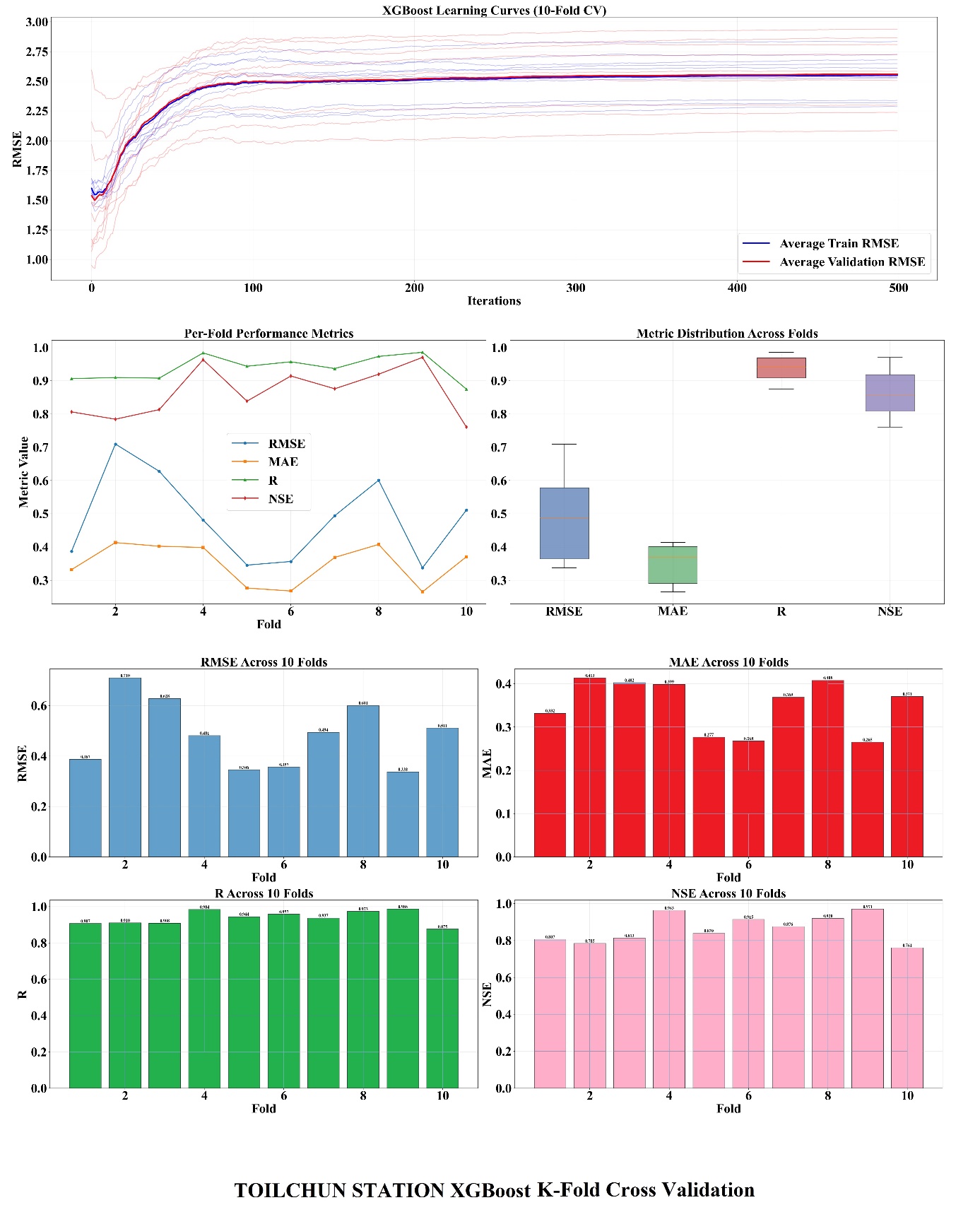
**

**Figure S2. 10-fold cross-validation results for COD prediction using the XGBoost model: Toilchun station.**


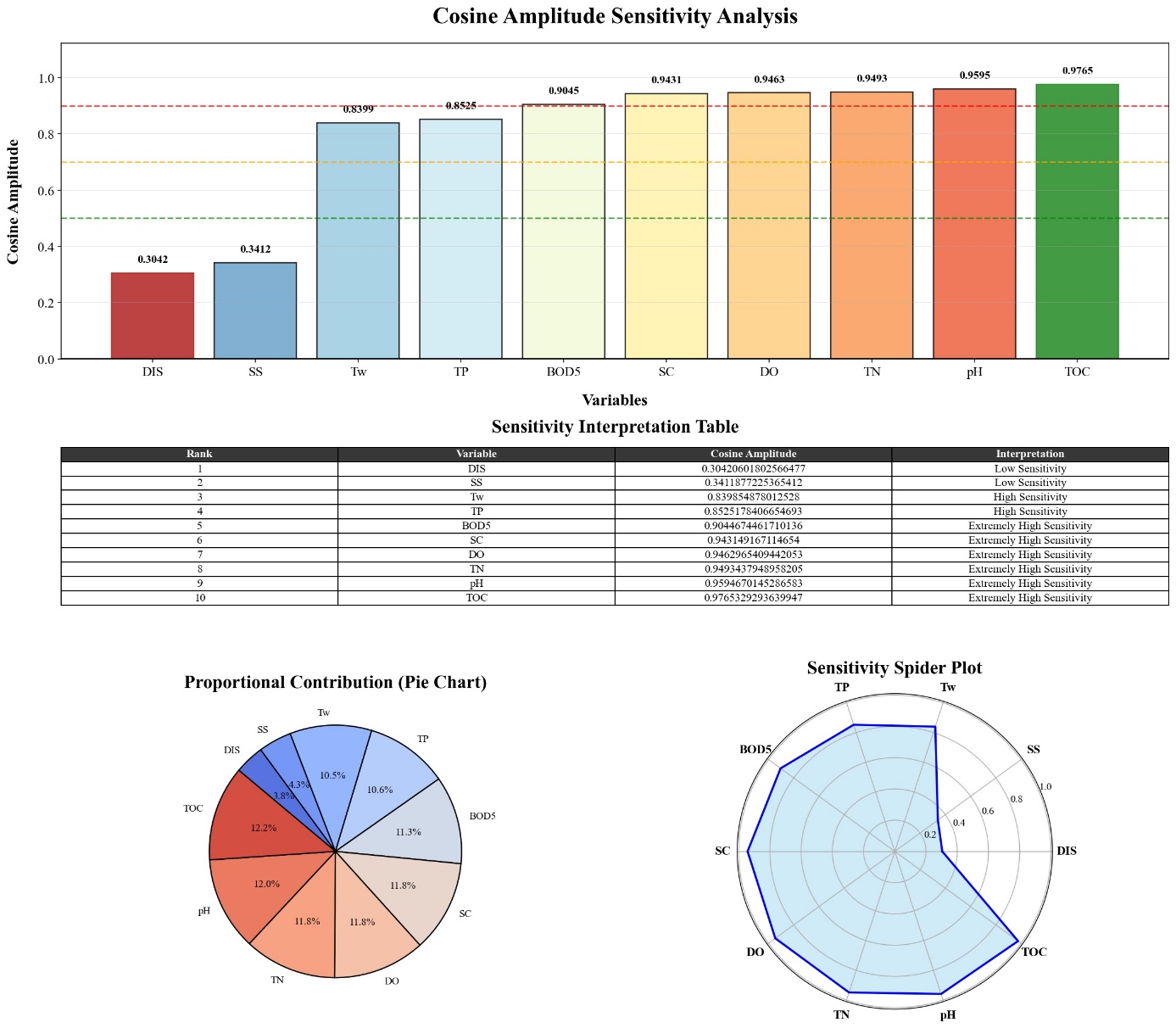


Figure S3. Cosine Amplitude Sensitivity Analysis: HWANGJI Station.


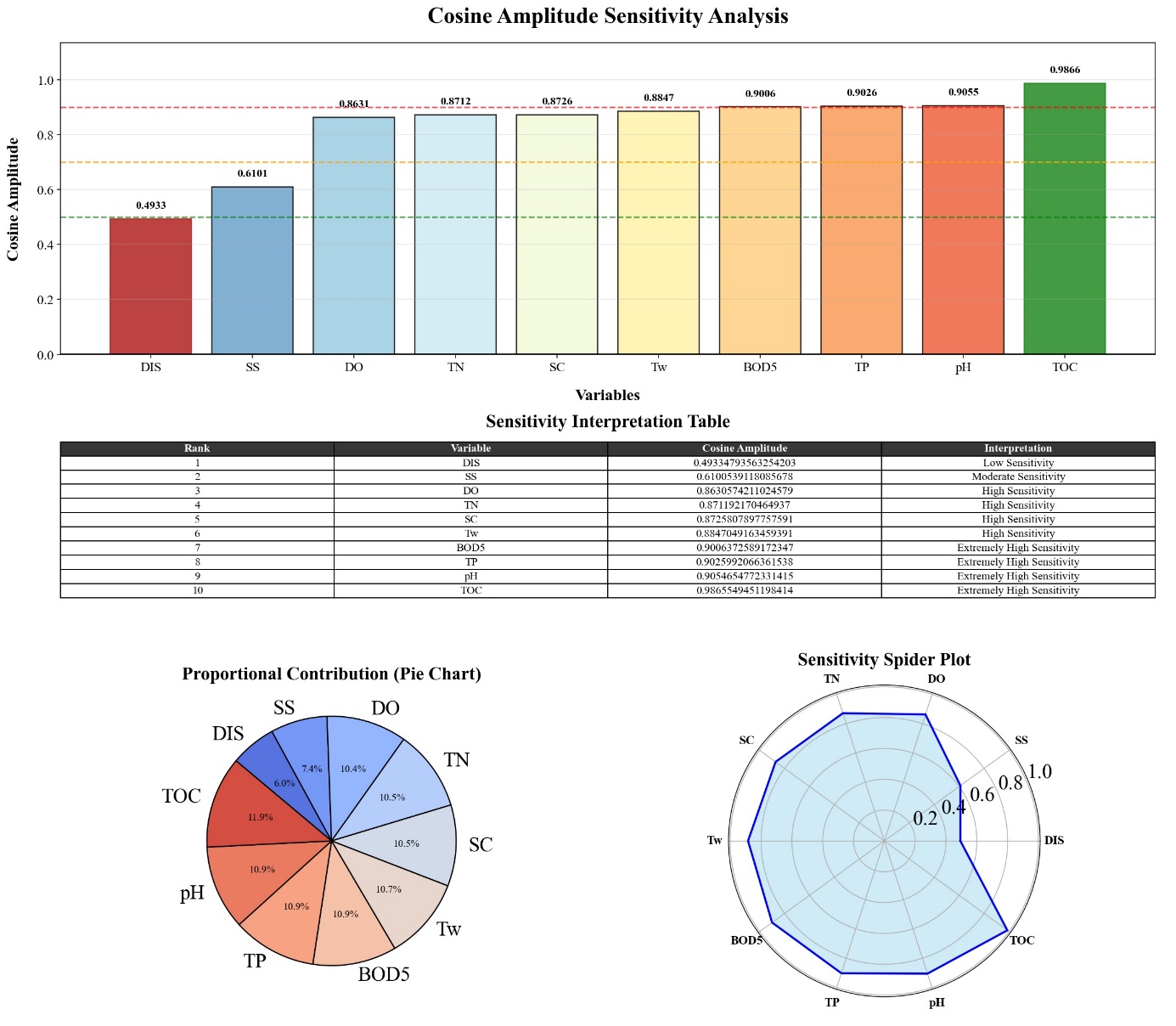


Figure S4. Cosine Amplitude Sensitivity Analysis: TOILCHUN Station.

**
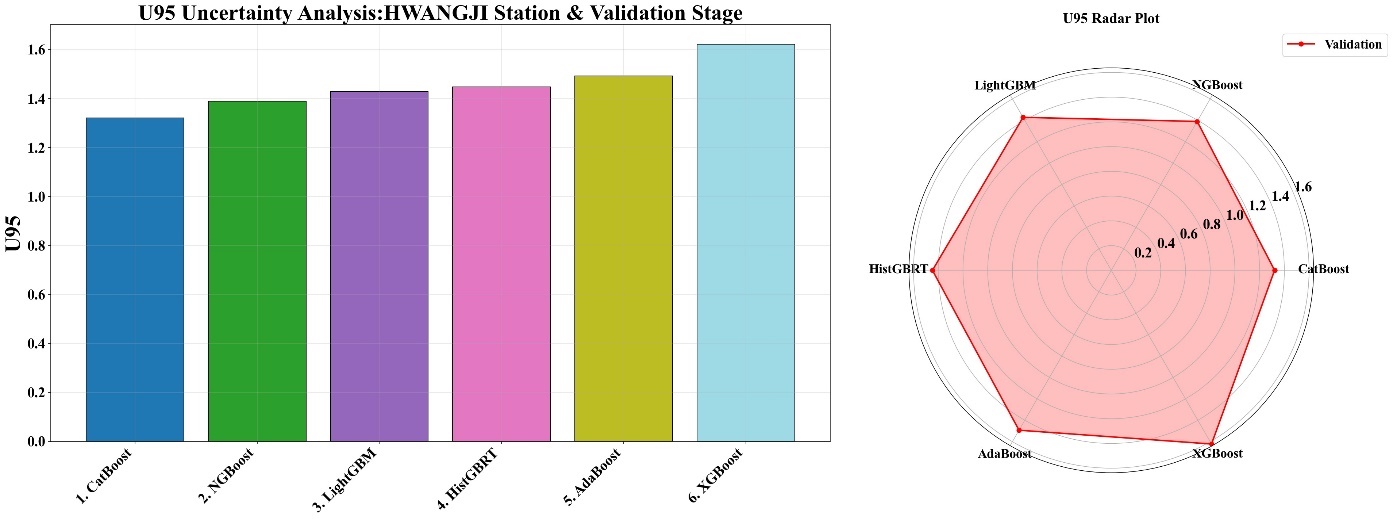
**

Figure S5. U₉₅ uncertainty analysis and models ranking: bar plot left panel and spider plot right panel: HWANGJI Station.

**
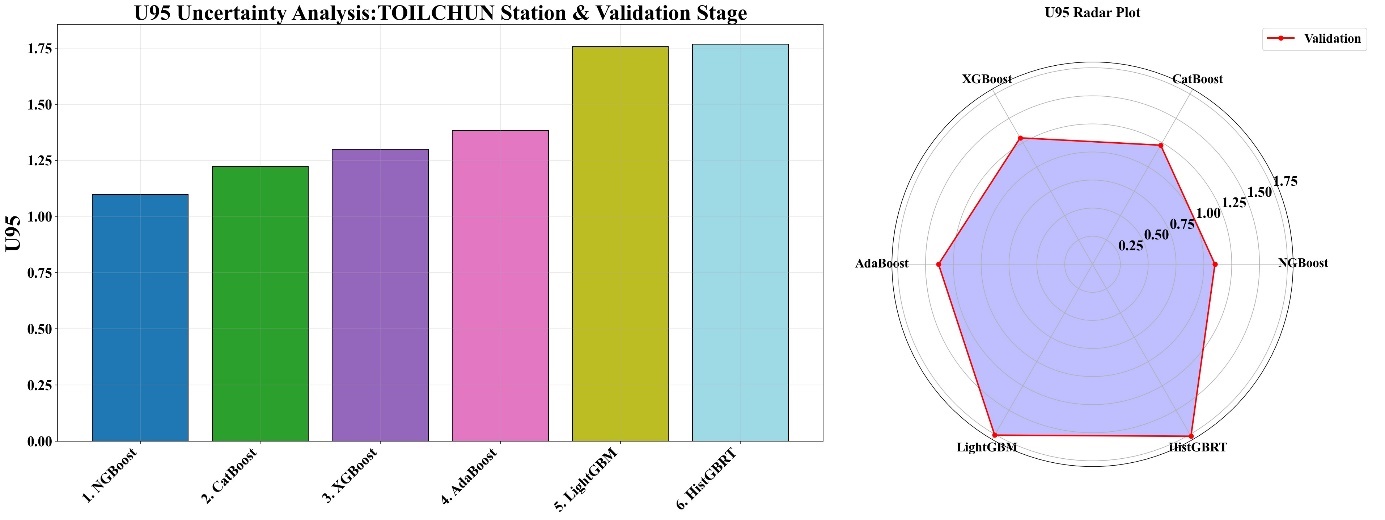
**

Figure S6. U₉₅ uncertainty analysis and models ranking: bar plot left panel and spider plot right panel: TOILCHUN Station.


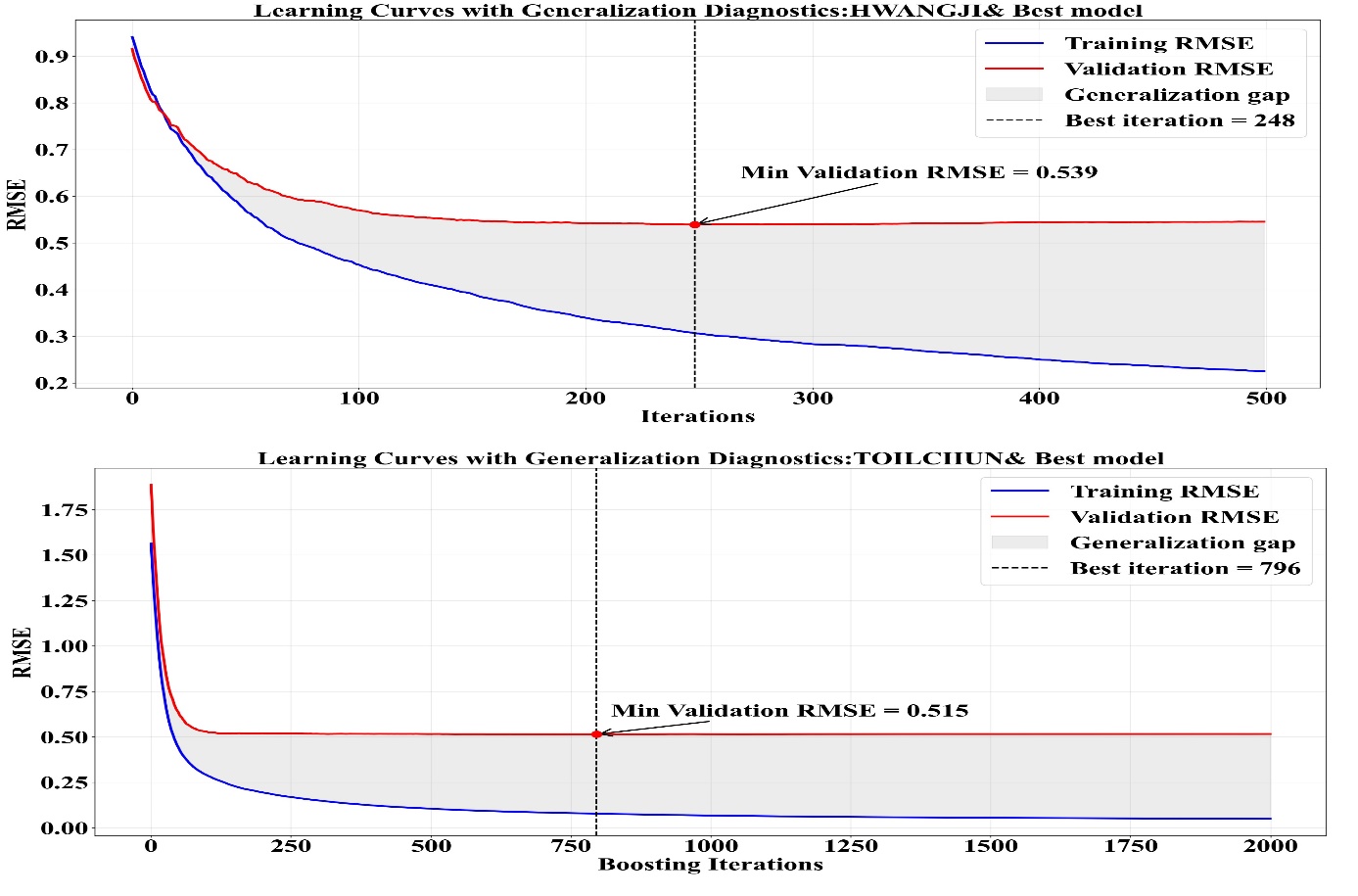


Figure S7. **Training and Validation Learning Curves with Generalization Diagnostics**


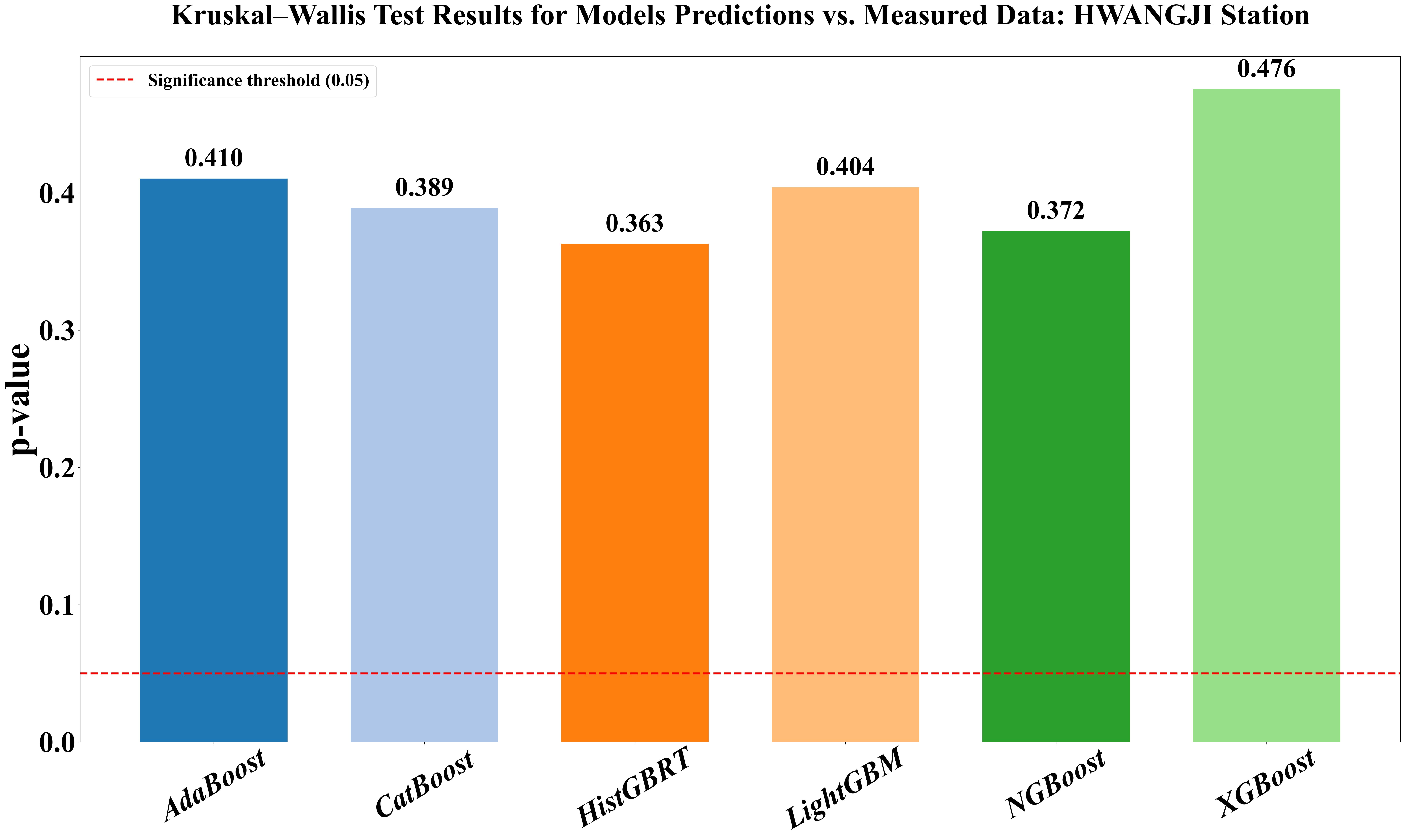


**Figure S8.** Statistical comparison of model performances at Hwangji station based on p-values from the Kruskal–Wallis test.


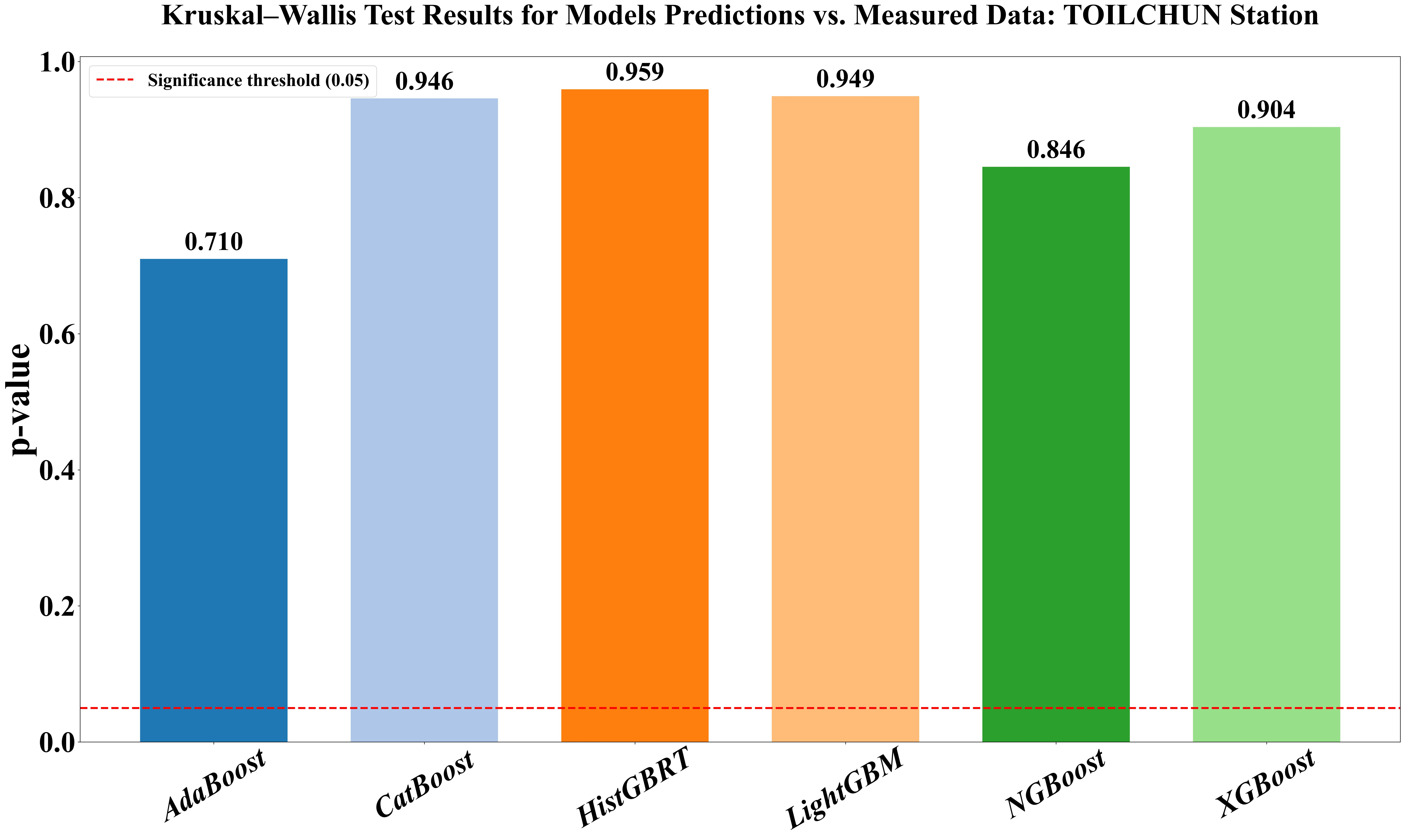


**Figure S9.** Statistical comparison of model performances at Toilchun station based on p-values from the Kruskal-Wallis test.
